# Supplementary material for: Integration of questionnaire-based risk factors improves polygenic risk scores for human coronary heart disease and type 2 diabetes
Source: Commun Biol. 2022 Feb 23;5:158. doi: 10.1038/s42003-021-02996-0 (PMC8866413; doi:10.1038/s42003-021-02996-0)
Supplement: Supplementary file 1 — Supplementary Information [file 42003_2021_2996_MOESM1_ESM.pdf]

**Supplementary Figure 1.** Receiver operator characteristic curves and AUCs for different risk models in the UK Biobank validation datasets for CHD (N = 242,687) and T2D (N = 121,113).

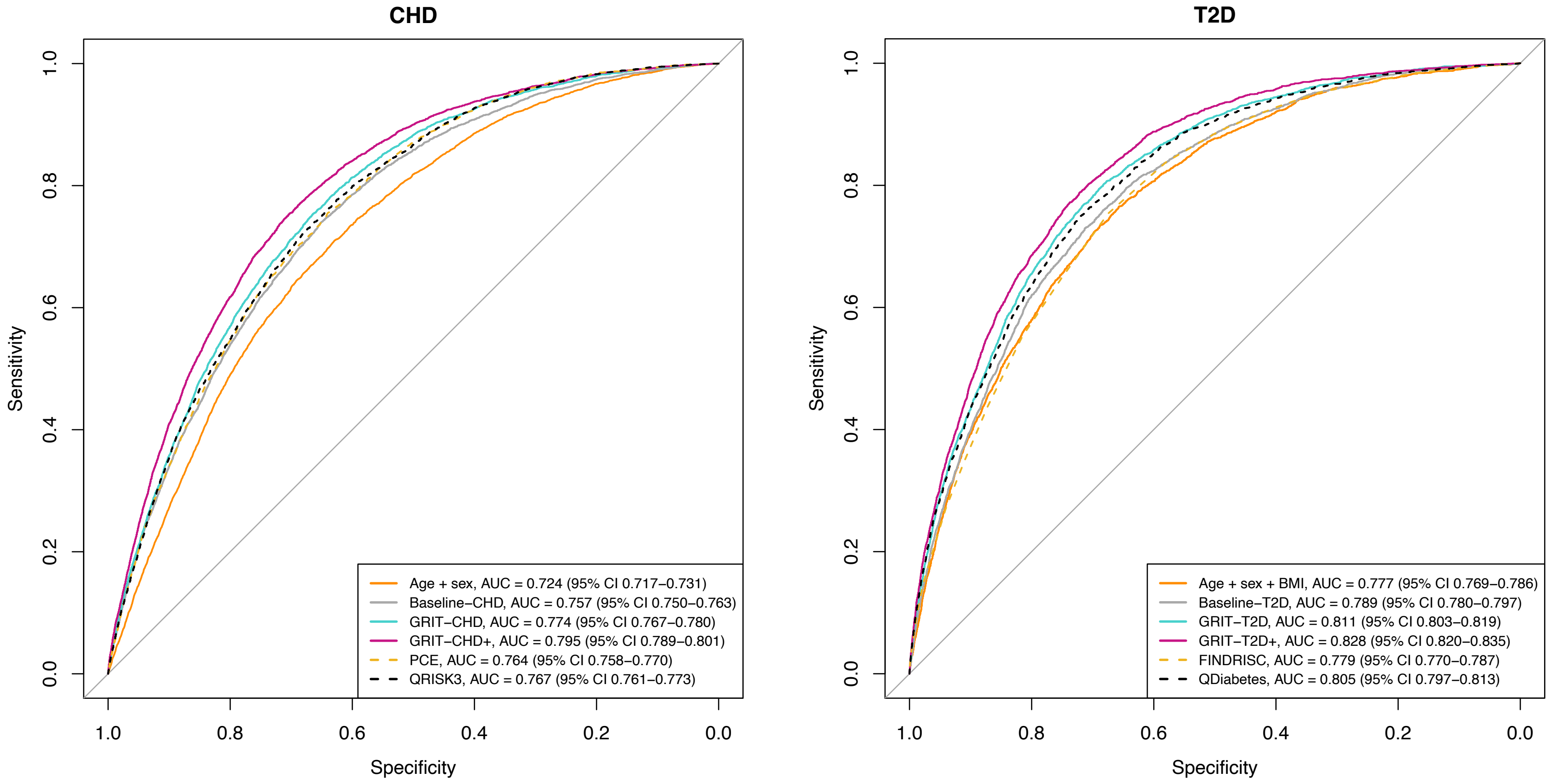

**Supplementary Figure 2.** Plots for goodness-of-fit separately for non-recalibrated and recalibrated model performance for GRIT-CHD, GRIT-CHD+, PCE and QRISK3 in the UK Biobank CHD dataset (N = 242,687).

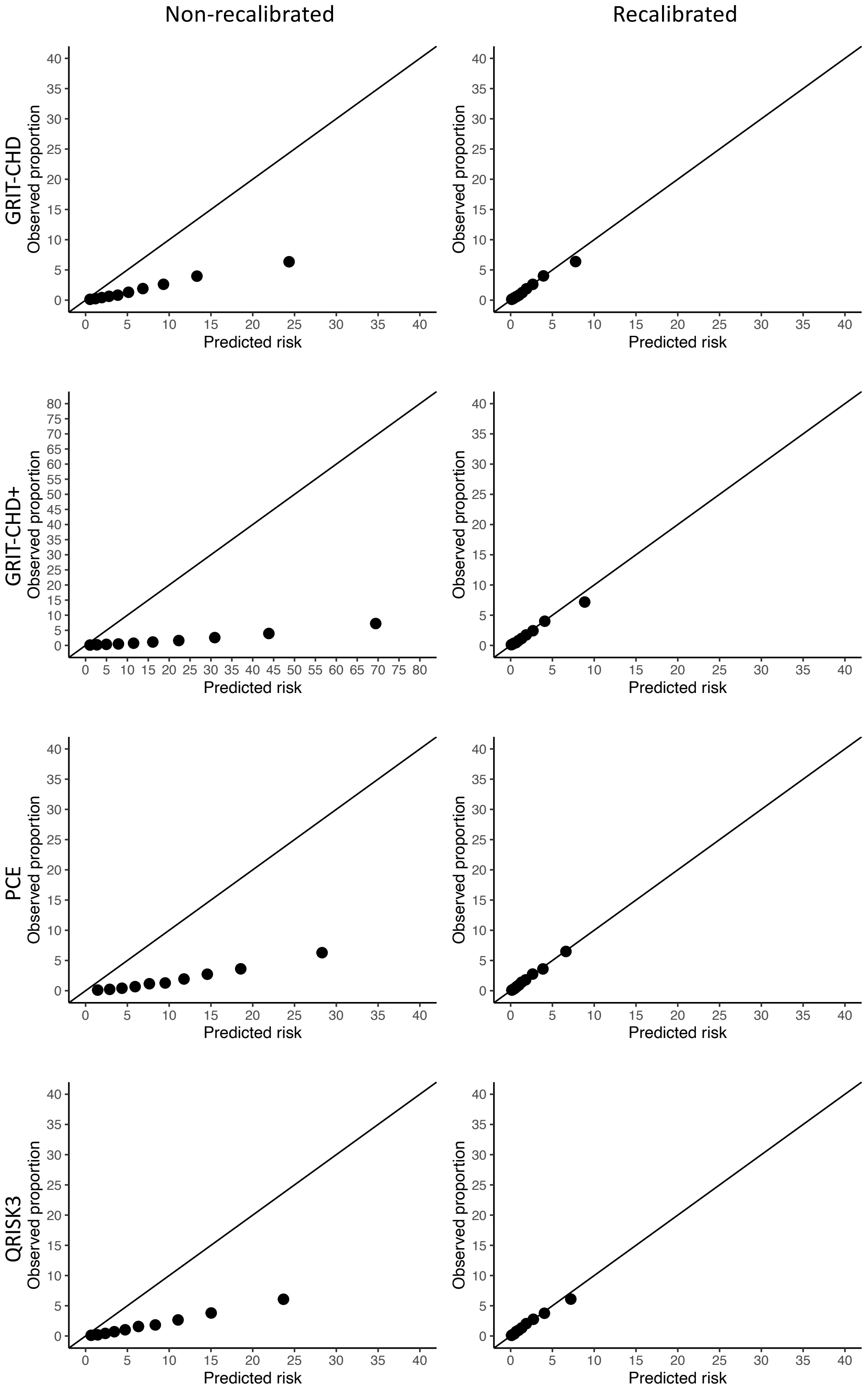

**Supplementary Figure 3.** Plots for goodness-of-fit separately for non-recalibrated and recalibrated model performance for GRIT-T2D, GRIT-T2D+ and QDiabetes in the UK Biobank T2D dataset (N = 121,113).

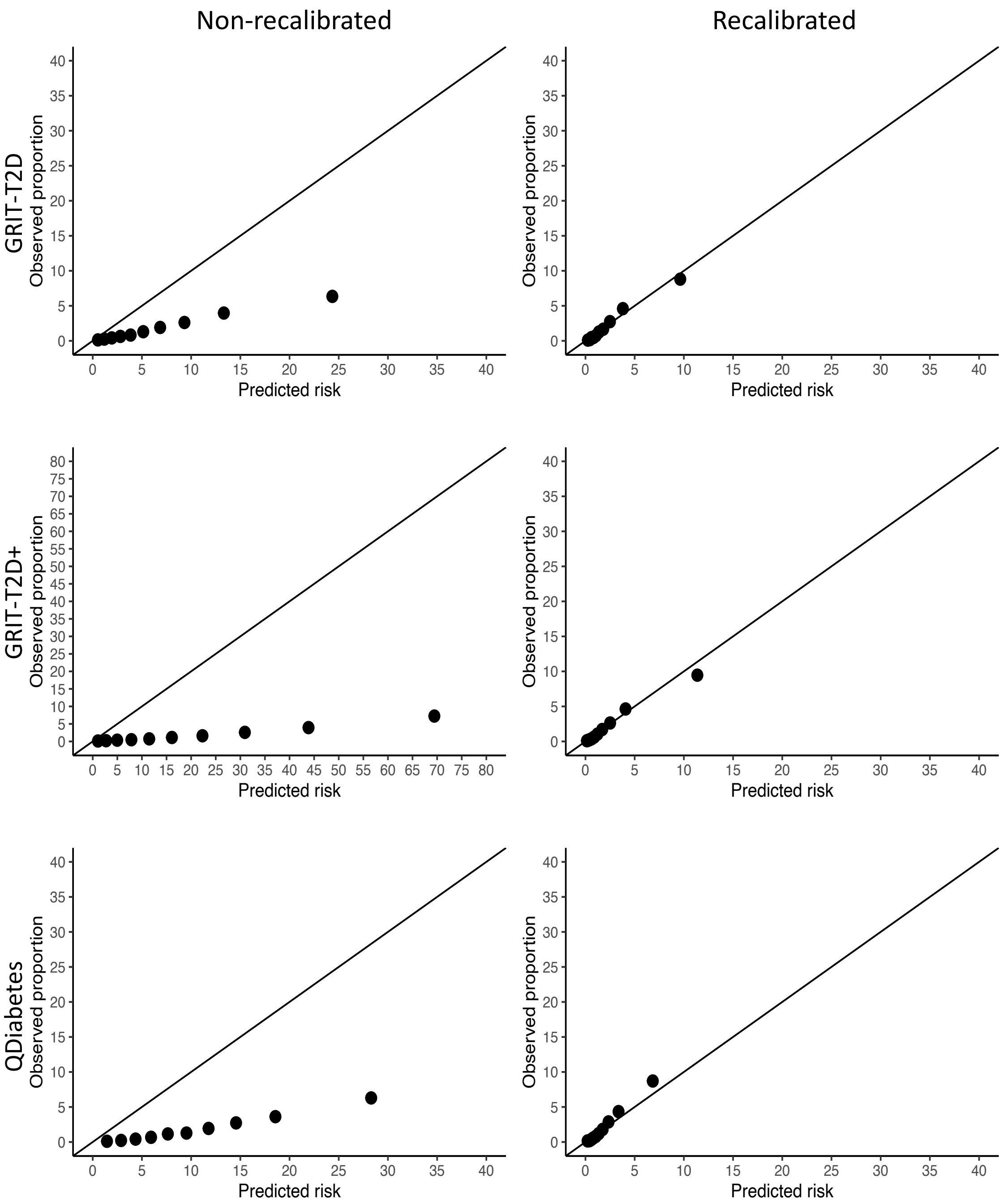

**Supplementary Figure 4.** Receiver operator characteristic curves and AUCs for different risk models in UK Biobank after recalibration for CHD (N = 242,687) and T2D (N = 121,113).

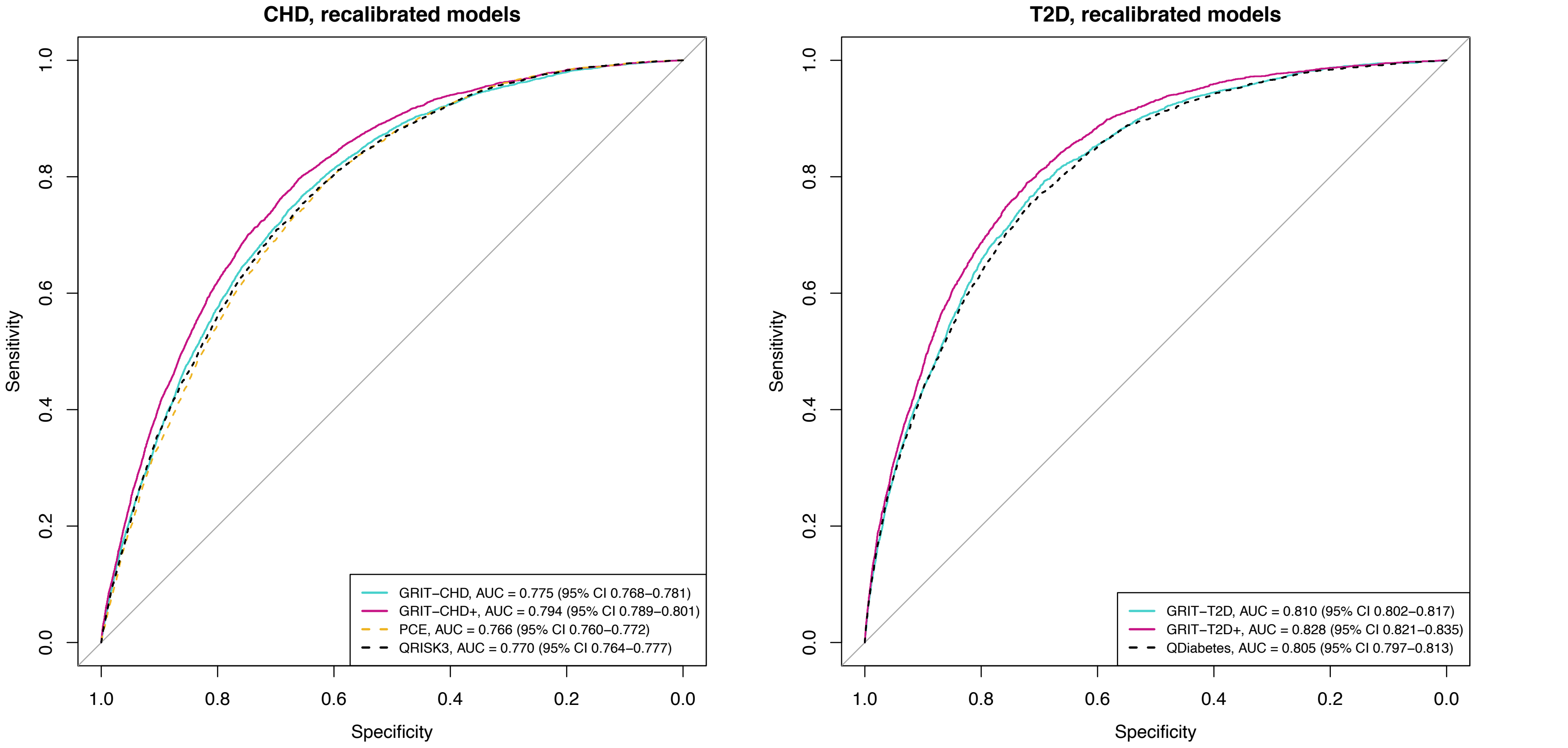

**Supplementary Figure 5.** Survival curves, 95% confidence intervals, and hazard ratios from Cox proportional hazards models in UK Biobank for the genomics-enhanced risk tools (GRIT-CHD, GRIT-CHD+, GRIT-T2D and GRIT-T2D+) for cumulative risk of **(a)** CHD (N = 242,687) and **(b)** T2D (N = 121,113) by categories based on GRIT distribution.

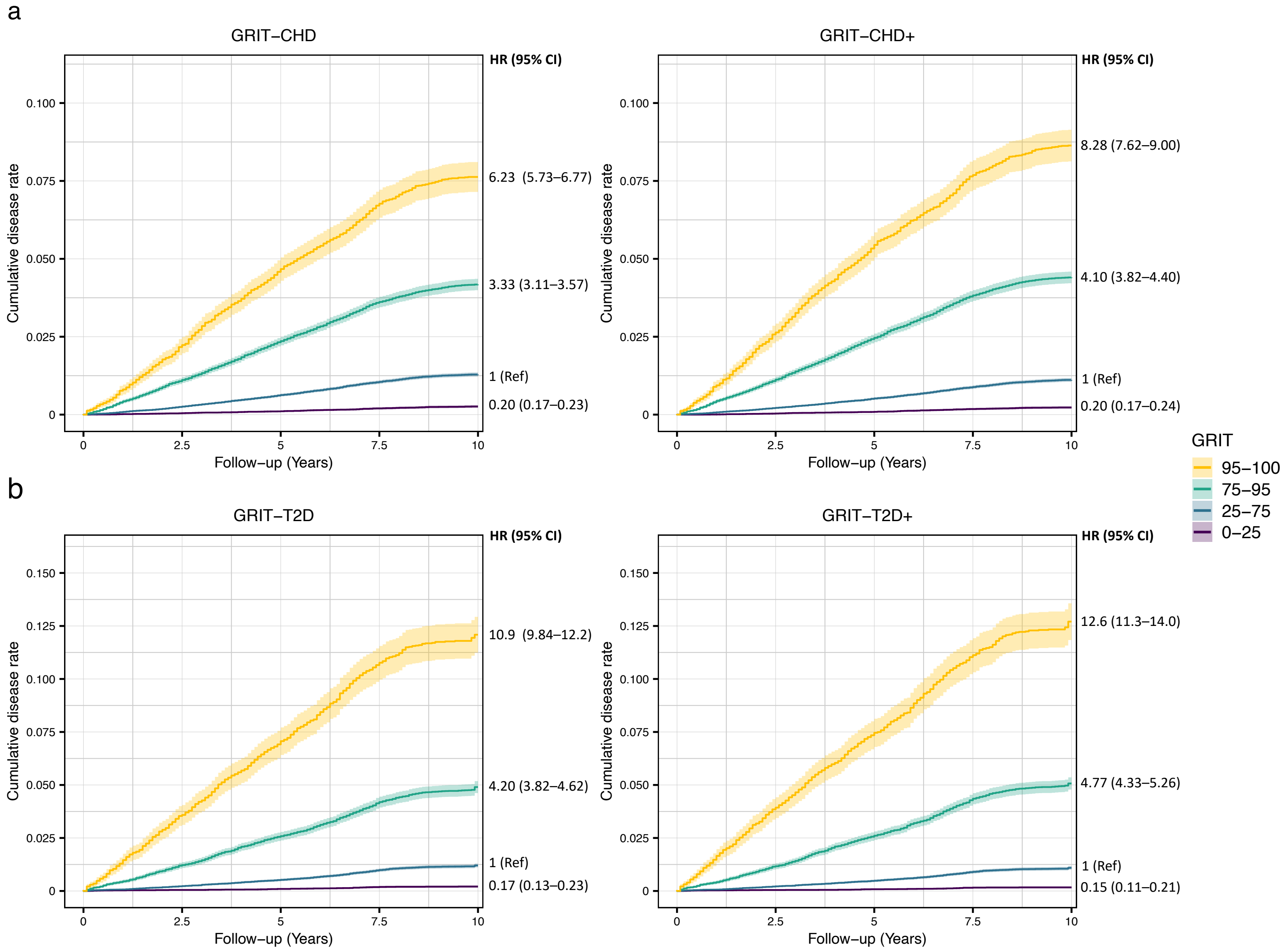

**Supplementary Figure 6.** Cross-predictive performance of the Genomics-enhanced RIsk Tools (GRIT). GRIT-CHD is tested in prediction of incident T2D, and GRIT-T2D in prediction of incident CHD. The sample size in CHD is slightly smaller (N = 242,565 with 4,467 incident cases) due to exclusions of individuals with missing values for TG needed for GRIT-T2D+. In T2D, the sample size becomes slightly smaller due to exclusions of missing values for LDL, resulting in a sample size of 120,951 with 2,542 incident cases. The Pearson correlation between the GRIT-CHD and GRIT-T2D was 0.42 in incident CHD analyses and 0.47 in incident T2D analyses. Similarly, the correlation between GRIT-CHD+ and GRIT-T2D+ was 0.54 in incident CHD analyses and 0.55 in incident T2D analyses. The Pearson correlations were statistically significant ($P < 2.2 \times 10^{-16}$ for all comparisons).

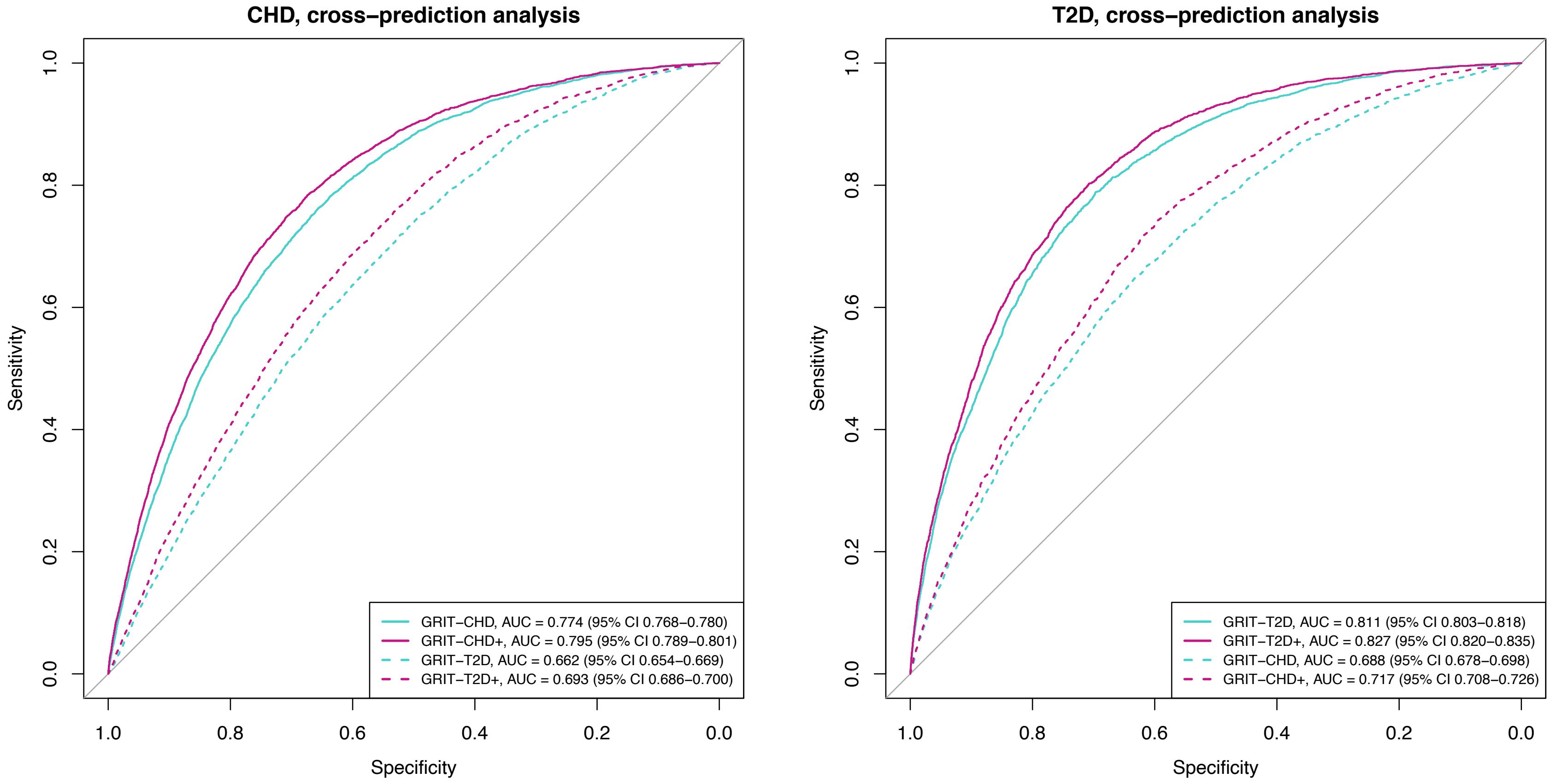

**Supplementary Table 1.** AUCs and ORs per SD (with 95% CI) for our PRSs in FinnGen and the UK Biobank separately for prevalent, incident, and all (prevalent and incident) disease cases in the full FinnGen dataset (N = 309,154) with 33,628 cases of CHD and 44,266 cases of T2D and the UK Biobank British ancestry subset (N = 343,672) with 18,698 cases of CHD and 24,192 cases of T2D. The estimates are from logistic regression models adjusted for year of birth, sex, and additionally ten first principal components of ancestry, batch, and genotyping array in FinnGen. The median follow-up time after enrollment in FinnGen was 15.3 years (interquartile range [IQR], 7.8–22.6) for CHD and 13.0 years (IQR 7.5–19.7) for T2D. The median follow-up after enrollment in UK Biobank was 10.7 years (IQR, 8.6–11.6) for CHD and 10.4 years (IQR, 8.3–11.3) for T2D.

| | Prevalent cases only | | Incident cases only | | All cases | |
|---|---|---|---|---|---|---|
| **PRS** | **AUC (95% CI)** | **OR per SD (95% CI)** | **AUC (95% CI)** | **OR per SD (95% CI)** | **AUC (95% CI)** | **OR per SD (95% CI)** |
| **$PRS_{CHD}$** | | | | | | |
| FinnGen | 0.869 (0.867–0.871) | 1.59 (1.57–1.62) | 0.913 (0.911–0.916) | 1.44 (1.41–1.47) | 0.871 (0.869–0.873) | 1.56 (1.53–1.58) |
| UK Biobank | 0.811 (0.808–0.815) | 1.77 (1.73–1.80) | 0.756 (0.751–0.761) | 1.61 (1.57–1.65) | 0.792 (0.789–0.795) | 1.72 (1.70–1.75) |
| **$PRS_{T2D}$** | | | | | | |
| FinnGen | 0.810 (0.808–0.813) | 1.59 (1.57–1.61) | 0.852 (0.849–0.855) | 1.52 (1.49–1.55) | 0.758 (0.756–0.761) | 1.59 (1.57–1.61) |
| UK Biobank | 0.725 (0.721–0.729) | 1.75 (1.72–1.78) | 0.669 (0.664–0.675) | 1.51 (1.48–1.54) | 0.708 (0.705–0.711) | 1.68 (1.65–1.70) |

**Supplementary Table 2.** The number of identified incident and prevalent CVD, CHD, and T2D cases by data source in the validation cohort, UK Biobank. The last column shows the number of events that were only identified using primary care data.

| Event | N cases in HES/mortality data (% of total N cases) | N cases in GP data (% of total N cases) | N cases in Nurse interview data (% of total N cases) | Total N cases | N cases in GP data only (% of total N cases) |
|---|---|---|---|---|---|
| Prevalent CVD (N = 343,672 before exclusions) | 14,312 (58.6%) | 6,986 (28.6%) | 21,196 (86.8%) | 24,415 | 784 (3.2%) |
| Prevalent T2D (N = 160,338 before exclusions) | 2,958 (38.3%) | 4,968 (64.3%) | 7,396 (95.8%) | 7,722 | 96 (1.2%) |
| Incident CHD (N = 242,687) | 4,377 (97.9%) | 1,014 (22.7%) | – | 4,469 | 92 (2.1%) |
| Incident T2D (N = 121,113) | 2,169 (85.3%) | 1,603 (63.0%) | – | 2,544 | 375 (14.7%) |

**Supplementary Table 3.** Absolute AUCs for measuring model discrimination in UK Biobank in all participants and stratified by sex, age, and BMI (only in T2D analyses).

| CHD | N total | N cases | PCE | QRISK3 | Baseline (CHD) | GRIT-CHD | GRIT-CHD+ |
|---|---|---|---|---|---|---|---|
| Overall | 242,687 | 4,469 | 0.764 (0.758-0.770) | 0.767 (0.761-0.773) | 0.757 (0.750-0.764) | 0.774 (0.768-0.780) | 0.795 (0.789-0.801) |
| Men | 105,439 | 3,272 | 0.695 (0.687-0.703) | 0.702 (0.694-0.710) | 0.688 (0.679-0.696) | 0.710 (0.702-0.718) | 0.740 (0.733-0.748) |
| Women | 137,248 | 1,197 | 0.744 (0.732-0.757) | 0.753 (0.740-0.765) | 0.718 (0.704-0.732) | 0.747 (0.734-0.761) | 0.771 (0.758-0.784) |
| Age < 55 | 101,508 | 963 | 0.801 (0.789-0.814) | 0.801 (0.788-0.814) | 0.777 (0.762-0.792) | 0.805 (0.791-0.819) | 0.822 (0.808-0.835) |
| Age ≥ 55 | 141,179 | 3,506 | 0.722 (0.714-0.730) | 0.727 (0.719-0.734) | 0.716 (0.707-0.724) | 0.730 (0.723-0.738) | 0.756 (0.749-0.764) |
| **T2D** | **N total** | **N cases** | **FINDRISC** | **QDiabetes** | **Baseline (T2D)** | **GRIT-T2D** | **GRIT-T2D+** |
| Overall | 121,113 | 2,581 | 0.779 (0.779-0.787) | 0.805 (0.797-0.813) | 0.788 (0.780-0.796) | 0.810 (0.803-0.818) | 0.827 (0.820-0.834) |
| Men | 55,898 | 1,532 | 0.765 (0.754-0.776) | 0.785 (0.774-0.796) | 0.779 (0.759-0.781) | 0.788 (0.777-0.799) | 0.804 (0.793-0.814) |
| Women | 65,215 | 1,012 | 0.799 (0.787-0.812) | 0.814 (0.802-0.826) | 0.792 (0.778-0.805) | 0.821 (0.809-0.833) | 0.843 (0.832-0.855) |
| Age < 55 | 46,238 | 568 | 0.798 (0.781-0.815) | 0.839 (0.823-0.856) | 0.829 (0.803-0.837) | 0.846 (0.830-0.861) | 0.862 (0.847-0.877) |
| Age ≥ 55 | 74,875 | 1,976 | 0.754 (0.744-0.764) | 0.774 (0.764-0.783) | 0.757 (0.747-0.767) | 0.780 (0.770-0.789) | 0.798 (0.789-0.807) |
| BMI < 30 | 94,006 | 1,100 | 0.748 (0.734-0.762) | 0.776 (0.763-0.789) | 0.751 (0.737-0.764) | 0.786 (0.773-0.799) | 0.808 (0.795-0.820) |
| BMI ≥ 30 | 27,107 | 1,444 | 0.659 (0.636-0.664) | 0.699 (0.686-0.713) | 0.689 (0.666-0.693) | 0.708 (0.695-0.721) | 0.734 (0.721-0.746) |

**Supplementary Table 4.** The continuous net reclassification improvement (NRI) and integrated discrimination improvement (IDI) between the genomics-enhanced risk tools and clinical risk scores (PCE for CHD and QDiabetes for T2D). The continuous NRI is defined as the sum of proportions of cases whose risk increase and non-cases whose risk decrease and the IDI as the difference in average predicted probabilities among cases and non-cases.

| | | **Continuous NRI [95% CI]** | **IDI [95% CI]** |
|---|---|---|---|
| GRIT-CHD vs PCE | Cases | 0.069 [0.039, 0.098] | – |
| | Non-cases | –0.022 [–0.026, –0.018] | – |
| | All | 0.047 [0.017, 0.076] | 0.0077 [0.0039, 0.012] |
| GRIT-CHD+ vs PCE | Cases | 0.21 [0.18, 0.24] | – |
| | Non-cases | –0.024 [–0.028, –0.020] | – |
| | All | 0.19 [0.16, 0.22] | 0.028 [0.024, 0.031] |
| GRIT-T2D vs QDiabetes | Cases | –0.022 [–0.061, 0.017] | – |
| | Non-cases | –0.0020 [–0.0077, 0.0037] | – |
| | All | –0.024 [–0.063, 0.015] | 0.0047 [0.00013, 0.0092] |
| GRIT-T2D+ vs QDiabetes | Cases | 0.11 [0.069, 0.15] | – |
| | Non-cases | 0.0020 [-0.0037, 0.0077] | – |
| | All | 0.11 [0.071, 0.15] | 0.023 [0.018, 0.028] |

**Supplementary Table 5.** Risk reclassification table and category-based net reclassification improvement (event NRI, nonevent NRI, overall NRI for incident CHD between genomics-enhanced risk tools (GRIT-CHD, GRIT-CHD+) and QRISK3 in UK Biobank at established 10-year clinical risk threshold of 10%.

| | | Upclassified to higher risk (%) | Both high risk (%) | Downclassified to lower risk (%) | Both low risk (%) | Category-based NRI [95% CI] |
|---|---|---|---|---|---|---|
| GRIT-CHD vs QRISK3 | Cases | 291 (6.0%) | 70 (1.6%) | 88 (2.0%) | 4,020 (90.4%) | 4.5 [3.7, 5.4] |
| | Non-cases | 3,005 (1.2%) | 548 (0.2%) | 933 (0.4%) | 233,732 (98.1%) | -0.8 (-0.9, -0.8] |
| | All | 3,296 (1.3%) | 618 (0.3%) | 1,021 (0.4%) | 237,752 (98.0%) | 3.7 [2.8, 4.6] |
| GRIT-CHD+ vs QRISK3 | Cases | 463 (11.0%) | 106 (2.4%) | 52 (1.1%) | 3,858 (86.5%) | 9.0 [8.1, 9.9] |
| | Non-cases | 4,620 (1.9%) | 888 (0.4%) | 593 (0.2%) | 232,117 (97.5%) | -1.7 [-1.8, -1.6] |
| | All | 5,083 (2.0%) | 994 (0.4%) | 645 (0.2%) | 235,975 (97.3%) | 7.3 [6.4, 8.1] |

**Supplementary Table 6.** The continuous net reclassification improvement (NRI) and integrated discrimination improvement (IDI) between the genomics-enhanced risk tools and QRISK3 in UK Biobank

| | | Continuous NRI [95% CI] | IDI [95% CI] |
|---|---|---|---|
| GRIT-CHD vs QRISK3 | Cases | 0.040 [0.0010, 0.069] | – |
| | Non-cases | –0.010 [–0.014, –0.0060] | – |
| | All | 0.030 [-0.0058, 0.059] | 0.0043 [0.00059, 0.0080] |
| GRIT-CHD+ vs QRISK3 | Cases | 0.15 [0.12, 0.18] | – |
| | Non-cases | 0.0018 [-0.022, 0.0058] | – |
| | All | 0.15 [0.12, 0.18] | 0.024 [0.021, 0.028] |

**Supplementary Table 7.** Cohorts and biobanks used to derive the models for CHD and T2D in FinnGen Data Freeze 7. *Hospital-based biobanks

| Cohort / Biobank | Final CHD dataset N individuals | Final T2D dataset N individuals |
|---|---|---|
| AURIA BIOBANK* | 2,149 | 2,505 |
| BIOBANK OF CENTRAL FINLAND* | 40 | 45 |
| BLOOD SERVICE BIOBANK | 564 | 597 |
| BOREALIS BIOBANK* | 2 | 4 |
| HELSINKI BIOBANK* | 870 | 1,115 |
| THL BIOBANK ATBC | 7,168 | 8,465 |
| THL BIOBANK BOTNIA | 5,734 | 5,666 |
| THL BIOBANK COROGENE | 388 | 2,739 |
| THL BIOBANK FINRISK 1992 | 3,932 | 4,032 |
| THL BIOBANK FINRISK 1997 | 5,589 | 5,790 |
| THL BIOBANK FINRISK 2002 | 5,338 | 5,738 |
| THL BIOBANK FINRISK 2007 | 3,701 | 4,300 |
| THL BIOBANK FINRISK 2012 | 3,647 | 4,213 |
| THL BIOBANK GENERISK | 4,394 | 4,766 |
| THL BIOBANK HEALTH 2000 | 5,064 | 5,383 |
| THL BIOBANK HEALTH 2011 | 606 | 617 |
| THL BIOBANK HHS | 3,239 | 3,289 |
| THL BIOBANK KUUSAMO | 151 | 191 |
| THL BIOBANK SUPER | 1,114 | 1,146 |
| THL BIOBANK T1D | 679 | 546 |
| THL BIOBANK TWINS | 7,509 | 8,012 |
| **SUM** | **61,878** | **69,159** |

**Supplementary Table 8.** Participant characteristic for included and excluded individuals in the CHD derivation analyses (FinnGen).

| Coronary heart disease analyses | Final CHD derivation set, N = 61,878 | | Excluded participants from CHD derivation set, N = 33,304 | | |
|---|---|---|---|---|---|
| | Men, N = 33,774 | Women, N = 28,104 | Men, N = 16,393 | Women, N = 16,911 | N missing |
| Age, mean ± SD | 53.4±10.7 | 51.3±10.8 | 56.6±11.3 | 54.1±11.9 | |
| Current smoker, *n* (%) | 15,923 (47.1) | 5,487 (19.5) | 3,612 (22.0) | 960 (5.7) | 19,695 |
| Any diabetes[1], *n* (%) | 1,400 (4.1) | 1,198 (4.3) | 3,026 (18.0) | 2,253 (13.0) | |
| Blood-pressure lowering medication, *n* (%) | 3,301 (9.8) | 3,961 (14.1) | 5,996 (36.6) | 5,040 (29.8) | |
| BMI, kg m-2, mean ± SD | 27.0±4.1 | 26.7±5.2 | 28.0±4.7 | 28.0±5.7 | 13,104 |

**Supplementary Table 9.** Participant characteristic for included and excluded individuals in the T2D derivation analyses (FinnGen).

| Type 2 diabetes analyses | Final T2D derivation set, N = 69,159 | | Excluded participants from T2D validation set, N = 26,023 | | |
|---|---|---|---|---|---|
| | Men, N = 38,861 | Women, N = 30,298 | Men, N = 11,306 | Women, N = 14,717 | N missing |
| Age, mean ± SD | 54.6±10.8 | 52.4±11.1 | 53.7±11.9 | 52.2±11.7 | |
| Current smoker, *n* (%) | 18,305 (47.1) | 5,876 (19.4) | 1,230 (10.9) | 571 (3.9) | 19,695 |
| Blood-pressure lowering medication, *n* (%) | 5,602 (14.0) | 5,377 (18.0) | 3,695 (33.0) | 3,624 (24.6) | |
| Lipid-lowering medication, *n* (%) | 2,735 (7.0) | 2,186 (7.2) | 2,168 (19.2) | 1,750 (11.9) | |
| BMI, kg m-2, mean ± SD | 27.0.9±4.0 | 26.7±5.1 | 28.6.9±5.2 | 28.4±6.3 | 13,104 |
| Prevalent CVD, *n* (%) | 4,652 (12.0) | 1,714 (5.7) | 2,096 (18.5) | 1,201 (8.2) | |
| Gestational diabetes, *n* (%) | n/a | 537 (1.8) | n/a | 571 (3.9) | |

**Supplementary Table 10.** Participant characteristic for included and excluded individuals in the CHD validation analyses (UK Biobank).

| Coronary heart disease analyses | Included participants in CHD validation set, N = 242,687 | | Excluded participants from CHD validation set, N = 100,985 | | |
|---|---|---|---|---|---|
| | Men, N = 105,439 | Women, N = 137,248 | Men, N = 53,662 | Women, N = 47,323 | N missing |
| Age, mean ± SD | 56.3±8.1 | 56.4±7.9 | 60.3±7.3 | 59.3±7.5 | |
| Current smoker, *n* (%) | 12,367 (11.7) | 11,554 (8.42) | 6,345 (11.9) | 4,347 (9.3) | 1,188 |
| Any diabetes[1], *n* (%) | 1,923 (1.8) | 1,323 (0.96) | 8,542 (16) | 4,681 (9.9) | |
| Blood-pressure lowering medication[2], *n* (%) | 12,638 (12.0) | 14,843 (10.8) | 26,900 (50.1) | 15,877 (33.6) | |
| BMI, kg m-2, mean ± SD | 27.4±4.0 | 26.7±4.9 | 28.7±4.5 | 27.1±5.5 | 1,084 |
| Family history of CHD, *n* (%) | 38,974 (37.0) | 59,867 (43.6) | 25,690 (47.9) | 24,727 (52.3) | |
| SBP, mmHg, mean ± SD | 143±18.4 | 137±20.2 | 144.3±18.7 | 140.1±20.3 | 319 |
| TC, mmol/L, mean ± SD | 5.8±1.01 | 6.0±1.1 | 4.8±1.1 | 5.4±1.1 | 16,072 |
| HDL-C, mmol/L, mean ± SD | 1.3±0.3 | 1.6±0.3 | 1.2±0.3 | 1.5±0.4 | 43,829 |
| LDL-C, mmol/L, mean ± SD | 3.7±0.8 | 3.8±0.8 | 3.0±0.8 | 3.3±0.9 | 16,691 |
| Smoking category | | | | | 1,188 |
| Non-smoker, *n* (%) | 55,482 (52.6) | 82,884 (60.4) | 22,358 (42.1) | 26,360 (56.4) | |
| Ex-smoker, *n* (%) | 37,590 (35.7) | 42,810 (31.2) | 24,396 (45.9) | 15,991 (34.2) | |
| Light smoker, *n* (%) | 1,163 (1.1) | 2,080 (1.5) | 527 (1.0) | 692 (1.5) | |
| Moderate smoker, *n* (%) | 7,771 (7.4) | 6,865 (5.0) | 3,925 (7.4) | 2,494 (5.3) | |
| Heavy smoker, *n* (%) | 3,433 (3.3) | 2,609 (1.9) | 1,893 (3.6) | 1,161 (2.5) | |
| Townsend index, mean ± SD | -1.6±2.9 | -1.7±2.8 | -1.3±3.1 | -1.4±3.0 | 411 |
| Rheumatoid arthritis, *n* (%) | 903 (0.9) | 2,342 (1.7) | 739 (1.4) | 1,147 (2.4) | |
| Atrial fibrillation, *n* (%) | 1,469 (1.4) | 811 (0.6) | 2,717 (5.1) | 919 (1.9) | |
| Chronic kidney disease, *n* (%) | 397 (0.4) | 748 (0.5) | 1,022 (1.9) | 973 (2.1) | |
| Migraine, *n* (%) | 4,000 (3.8) | 12,094 (8.8) | 2,226 (4.2) | 4,292 (9.1) | |
| Corticosteroid medication, *n* (%) | 978 (0.9) | 1,431 (1.0) | 804 (1.5) | 733 (1.6) | |
| SLE, *n* (%) | 36 (0.03) | 269 (0.2) | 31 (0.06) | 146 (0.3) | |
| Atypical antipsychotics, *n* (%) | 288 (0.3) | 293 (0.2) | 195 (0.4) | 168 (0.4) | |
| Severe mental illness, *n* (%) | 1,177 (1.1) | 2,166 (1.6) | 771 (.4) | 886 (1.9) | |
| Diagnosis of or treatment for erectile dysfunction, *n* (%) | 814 (0.8) | n/a | 1,104 (2.1) | n/a | |

**Supplementary Table 11.** Participant characteristic for included and excluded individuals in the T2D validation analyses (UK Biobank). The missing counts in the T2D dataset are calculated from the British ancestry dataset with primary care data available (N = 160,338).

| **Type 2 diabetes analyses** | **Included participants in T2D validation set, N = 121,113** | | **Excluded participants from T2D validation set, N = 222,559** | | |
|---|---|---|---|---|---|
| | **Men, N = 55,898** | **Women, N = 65,215** | **Men, N = 103,203** | **Women, N = 119,356** | **N missing** |
| Age, mean ± SD | 57.3±8.1 | 56.9±7.9 | 57.8±8.1 | 57.3±7.9 | |
| Current smoker, *n* (%) | 6,294 (11.3) | 5,542 (8.5) | 12,418 (12.1) | 10,359 (8.7) | 532 |
| Blood-pressure lowering medication, *n* (%) | 12,034 (21.5) | 9,620 (14.8) | 27,504 (26.7) | 21,100 (17.7) | |
| Lipid-lowering medication, *n* (%) | 10,196 (18.0) | 6,052 (9.3) | 24,669 (24.0) | 15,440 (13.0) | |
| BMI, kg m-2, mean ± SD | 27.6±4.0 | 26.8±4.86 | 28.0±4.3 | 27.2±5.3 | 497 |
| Prevalent CVD, *n* (%) | 5,161 (9.2) | 2,554 (3.9) | 11,221 (11.0) | 5,479 (4.6) | |
| Gestational diabetes, *n* (%) | n/a | 242 (0.4) | n/a | 543 (0.5) | |
| Family history of diabetes, *n* (%) | 10,208 (18.3) | 13,671 (21) | 21,294 (20.6) | 26,421 (22.1) | |
| SBP, mmHg, mean ± SD | 143.4±18.5 | 137.5±20.2 | 143.2±18.5 | 137.7±20.3 | 172 |
| HDL-C, mmol/L, mean ± SD | 1.3±0.3 | 1.6±0.4 | 1.3±0.3 | 1.6±0.4 | 20,713 |
| TG, mmol/L, mean ± SD | 2.0±1.1 | 1.5±0.8 | 2.0±1.2 | 1.6±0.9 | 7,444 |
| Smoking category | | | | | 532 |
| Non-smoker, *n* (%) | 28,267 (50.6) | 39,072 (59.9) | 49573 (48.3) | 70,172 (59.1) | |
| Ex-smoker, *n* (%) | 21,337 (38.2) | 20,601 (31.6) | 40649 (39.6) | 38,200 (32.2) | |
| Light smoker, *n* (%) | 575 (1.0) | 954 (1.5) | 1115 (1.1) | 1,818 (1.5) | |
| Moderate smoker, *n* (%) | 3,969 (7.1) | 3,336 (5.1) | 7727 (7.5) | 6,023 (5.1) | |
| Heavy smoker, *n* (%) | 1,750 (3.1) | 1,252 (1.9) | 3576 (3.5) | 2,518 (2.1) | |
| Townsend index, mean ± SD | -1.7±2.9 | -1.7±2.8 | -1.5±3.0 | -1.6±2.9 | 231 |
| Corticosteroid medication, *n* (%) | 573 (1.0) | 716 (1.1) | 1209 (1.1) | 1,448 (1.2) | |
| Atypical antipsychotics, *n* (%) | 156 (0.3) | 122 (0.2) | 327 (0.3) | 339 (0.3) | |
| Learning disabilities, *n* (%) | 15 (0.03) | 6 (0.009) | 23 (0.02) | 15 (0.01) | |
| Bipolar affective disorder or schizophrenia, *n* (%) | 289 (0.5) | 268 (0.4) | 535 (0.5) | 534 (0.4) | |
| Polycystic ovary syndrome, *n* (%) | n/a | 215 (0.3) | n/a | 348 (0.3) | |
| HbA1c, mmol/mol, mean ± SD | 5.4±0.5 (N missing but not excluded 2,501) | 5.4±0.4 (2,867) | 5.5±0.7 (4,977) | 5.4±0.6 (5,829) | |
| Physical activity ≥4h week, *n* (%) | 16,762 (30.0) | 21,870 (33.4) | 29,062 (31.5) | 34,032 (33.4) | 13,443 |
| Daily consumption of vegetables, fruits, or berries, *n* (%) | 54,572 (97.6) | 64,571 (99.0) | 100,134 (97.0) | 117,805 (98.7) | |

**Supplementary Table 12.** Baseline survival, beta coefficients, and mean component of risk tools for CHD derived in FinnGen (women).

| CHD models for women | Baseline survival | Beta coefficient | | | | | | | | | | Mean |
|---|---|---|---|---|---|---|---|---|---|---|---|---|
| | | PRS (scaled) | Age | Smoking | BMI | Antihypertensive | Diabetes | Family history of CHD | SBP | LDL | HDL | |
| Age (+ sex) | 0.98495 | – | 0.10759 | – | – | – | – | – | – | – | – | 5.51674 |
| + PRS | 0.98648 | 0.49024 | 0.11041 | | | | | | | | | 5.67772 |
| + Smoking | 0.98587 | – | 0.11469 | 0.82826 | – | – | – | – | – | – | – | 6.04242 |
| + BMI, kg/m^2 | 0.98513 | – | 0.10473 | – | 0.03428 | – | – | – | – | – | – | 6.28431 |
| + Antihypertensive | 0.98496 | – | 0.10060 | – | – | 0.58665 | – | – | – | – | – | 5.24145 |
| + Diabetes | 0.98527 | – | 0.10297 | – | – | – | 1.24358 | – | – | – | – | 5.33299 |
| + Family history of CHD | 0.98495 | – | 0.10759 | – | – | – | – | 0.37156 | – | – | – | 5.51674 |
| + SBP, mmHg | 0.98495 | – | 0.10759 | – | – | – | – | – | 0.00995 | – | – | 5.51674 |
| + LDL, mmol/L | 0.98495 | – | 0.10759 | – | – | – | – | – | – | 0.27763 | – | 5.51674 |
| + HDL, mmol/L | 0.98495 | – | 0.10759 | – | – | – | – | – | – | – | -0.86750 | 5.51674 |
| Age (+ sex) + PRS (Baseline model for CHD) | 0.98648 | 0.49024 | 0.11041 | – | – | – | – | – | – | – | – | 5.67772 |
| GRIT-CHD without PRS | 0.98623 | – | 0.10467 | 0.83443 | 0.02011 | 0.42210 | 1.07086 | 0.37156 | – | – | – | 6.15072 |
| GRIT-CHD+ without PRS | 0.98623 | – | 0.10467 | 0.83443 | 0.02011 | 0.42210 | 1.07086 | 0.37156 | 0.00995 | 0.27763 | -0.86750 | 6.15072 |
| GRIT–CHD | 0.98743 | 0.44886 | 0.10759 | 0.80069 | 0.01771 | 0.36356 | 1.03051 | 0.37156 | – | – | – | 6.25557 |
| GRIT-CHD+ | 0.98743 | 0.44886 | 0.10759 | 0.80069 | 0.01771 | 0.36356 | 1.03051 | 0.37156 | 0.00995 | 0.27763 | -0.86750 | 6.25557 |

**Supplementary Table 13.** Baseline survival, beta coefficients, and mean component of risk tools for CHD derived in FinnGen (men).

| CHD models for men | Baseline survival | Beta coefficient | | | | | | | | | | Mean |
|---|---|---|---|---|---|---|---|---|---|---|---|---|
| | | PRS (scaled) | Age | Smoking | BMI | Antihypertensive | Diabetes | Family history of CHD | SBP | LDL | HDL | |
| Age (+ sex) | 0.92194 | – | 0.080052 | – | – | – | – | – | – | – | – | 4.27152 |
| + PRS | 0.92706 | 0.42207 | 0.82958 | – | – | – | – | – | – | – | – | 4.41496 |
| + Smoking | 0.92636 | – | 0.078701 | 0.66758 | – | – | – | – | – | – | – | 4.51412 |
| + BMI, kg/m^2 | 0.92252 | – | 0.079697 | – | 0.032430 | – | – | – | – | – | – | 5.14743 |
| + Antihypertensive | 0.92182 | – | 0.079477 | – | – | 0.096333 | – | – | – | – | – | 4.25022 |
| + Diabetes | 0.92220 | – | 0.078900 | – | – | – | 0.67221 | – | – | – | – | 4.23805 |
| + Family history of CHD | 0.92193 | – | 0.107590 | – | – | – | – | 0.37156 | – | – | – | 4.27152 |
| + SBP, mmHg | 0.92655 | – | 0.107590 | – | – | – | – | – | 0.00995 | – | – | 4.27152 |
| + LDL, mmol/L | 0.92655 | – | 0.107590 | – | – | – | – | – | – | 0.27763 | – | 4.27152 |
| + HDL, mmol/L | 0.92655 | – | 0.107590 | – | – | – | – | – | – | – | -0.86750 | 4.27152 |
| Age (+ sex) + PRS (Baseline model for CHD) | 0.92705 | 0.42206 | 0.082958 | – | – | – | – | – | – | – | – | 4.41496 |
| GRIT-CHD without PRS | 0.92766 | – | 0.076337 | 0.74915 | 0.029390 | 0.21737 | 0.66507 | 0.37156 | – | – | – | 5.26909 |
| GRIT-CHD+ without PRS | 0.92766 | – | 0.076337 | 0.74915 | 0.029390 | 0.21737 | 0.66507 | 0.37156 | 0.00995 | 0.27763 | -0.86750 | 5.26909 |
| GRIT-CHD | 0.93219 | 0.41083 | 0.079452 | 0.724345 | 0.029283 | 0.19810 | 0.63650 | 0.37156 | – | – | – | 5.40634 |
| GRIT-CHD+ | 0.93219 | 0.41083 | 0.079452 | 0.724345 | 0.029283 | 0.19810 | 0.63650 | 0.37156 | 0.00995 | 0.27763 | -0.86750 | 5.40634 |

**Supplementary Table 14.** Baseline survival, beta coefficients, and mean component of risk tools for T2D derived in FinnGen (women).

| T2D models for women | Baseline survival | Beta coefficient | | | | | | | | | | | | Mean |
|---|---|---|---|---|---|---|---|---|---|---|---|---|---|---|
| | | PRS (scaled) | Age | BMI | Smoking | Antihypertensive | Statin | CVD | GDM | Family history of DM | SBP | TG | HDL | |
| Age (+ sex) | 0.94172 | – | 0.05083 | – | – | – | – | – | – | – | – | – | – | 2.66467 |
| + PRS | 0.94738 | 0.48191 | 0.05245 | – | – | – | – | – | – | – | – | – | – | 2.75621 |
| + BMI | 0.95352 | – | 0.42100 | 0.13215 | – | – | – | – | – | – | – | – | – | 5.72956 |
| + Smoking | 0.94243 | – | 0.05324 | – | 0.41739 | – | – | – | – | – | – | – | – | 2.88339 |
| + Antihypertensive | 0.94371 | – | 0.03138 | – | – | 0.92230 | – | – | – | – | – | – | – | 2.05812 |
| + Statin | 0.94198 | – | 0.04457 | – | – | – | 0.62241 | – | – | – | – | – | – | 2.38160 |
| + CVD | 0.94195 | – | 0.04664 | – | – | – | – | 0.52580 | – | – | – | – | – | 2.47483 |
| + GDM | 0.94280 | – | 0.05434 | – | – | – | – | – | 1.65637 | – | – | – | – | 2.87810 |
| + Family history of diabetes | 0.94172 | – | 0.05083 | – | – | – | – | – | – | 0.37400 | – | – | – | 2.66468 |
| + SBP, mmHg | 0.94172 | – | 0.05083 | – | – | – | – | – | – | – | 0.00701 | – | – | 2.66468 |
| + TG, mmol/L | 0.94172 | – | 0.05083 | – | – | – | – | – | – | – | – | 0.15718 | – | 2.66468 |
| + HDL, mmol/L | 0.94172 | – | 0.05083 | – | – | – | – | – | – | – | – | – | -0.78135 | 2.66468 |
| Age (+ sex) + PRS + BMI (Baseline model for T2D) | 0.95817 | 0.46555 | 0.04453 | 0.13175 | – | – | – | – | – | – | – | – | – | 5.85241 |
| GRIT–T2D without PRS | 0.95573 | – | 0.03616 | 0.12731 | 0.51732 | 0.50950 | 0.32830 | 0.24644 | 1.18688 | 0.37400 | – | – | – | 5.53901 |
| GRIT-T2D+ without PRS | 0.95573 | – | 0.03616 | 0.12731 | 0.51732 | 0.50950 | 0.32830 | 0.24644 | 1.18688 | 0.37400 | 0.00701 | 0.15718 | -0.78135 | 5.53901 |
| GRIT–T2D | 0.95991 | 0.44861 | 0.03876 | 0.12716 | 0.49738 | 0.48189 | 0.33099 | 0.23834 | 1.08052 | 0.37400 | – | – | – | 5.66610 |
| GRIT-T2D+ | 0.95991 | 0.44861 | 0.03876 | 0.12716 | 0.49738 | 0.48189 | 0.33099 | 0.23834 | 1.08052 | 0.37400 | 0.00701 | 0.15718 | -0.78135 | 5.66610 |

**Supplementary Table 15.** Baseline survival, beta coefficients, and mean of risk tools for T2D derived in FinnGen (men).

| T2D models for men | Baseline survival | Beta coefficient | | | | | | | | | | | | Mean |
|---|---|---|---|---|---|---|---|---|---|---|---|---|---|---|
| | | PRS (scaled) | Age | BMI | Smoking | Antihypertensive | Statin | CVD | GDM (women only) | Family history of DM | SBP | TG | HDL | |
| Age (+ sex) | 0.94173 | – | 0.05083 | – | – | – | – | – | – | – | – | – | – | 2.66468 |
| + PRS | 0.91211 | 0.45004 | 0.05017 | – | – | – | – | – | – | – | – | – | – | 2.81357 |
| + BMI | 0.91860 | – | 0.05093 | 0.14838 | – | – | – | – | – | – | – | – | – | 6.79505 |
| + Smoking | 0.90503 | – | 0.05017 | – | 0.06152 | – | – | – | – | – | – | – | – | 2.76950 |
| + Antihypertensive | 0.90982 | – | 0.04032 | – | – | 0.84609 | – | – | – | – | – | – | – | 2.32450 |
| + Statin | 0.90686 | – | 0.04581 | – | – | – | 0.67139 | – | – | – | – | – | – | 2.54959 |
| + CVD | 0.90818 | – | 0.04377 | – | – | – | – | 0.57912 | – | – | – | – | – | 2.46049 |
| + GDM (women only) | 0.94173 | – | 0.05083 | – | – | – | – | – | – | – | – | – | – | 2.66468 |
| + Family history of diabetes | 0.94172 | – | 0.05083 | – | – | – | – | – | – | 0.37400 | – | – | – | 2.66468 |
| + SBP, mmHg | 0.94172 | – | 0.05083 | – | – | – | – | – | – | – | 0.00701 | – | – | 2.66468 |
| + TG, mmol/L | 0.94172 | – | 0.05083 | – | – | – | – | – | – | – | – | 0.15718 | – | 2.66468 |
| + HDL, mmol/L | 0.94172 | – | 0.05083 | – | – | – | – | – | – | – | – | – | -0.78135 | 2.66468 |
| Age (+ sex) + PRS + BMI (Baseline model for T2D) | 0.93344 | 0.45878 | 0.05306 | 0.15171 | – | – | – | – | – | – | – | – | – | 6.99726 |
| GRIT–T2D without PRS | 0.92766 | – | 0.04023 | 0.14181 | 0.26852 | 0.48876 | 0.20745 | 0.33755 | – | 0.37400 | – | – | – | 6.28519 |
| GRIT-T2D+ without PRS | 0.92766 | – | 0.04023 | 0.14181 | 0.26852 | 0.48876 | 0.20745 | 0.33755 | – | 0.37400 | 0.00701 | 0.15718 | -0.78135 | 6.28519 |
| GRIT–T2D | 0.93424 | 0.45201 | 0.04275 | 0.14444 | 0.24392 | 0.50134 | 0.17777 | 0.30528 | – | 0.37400 | – | – | – | 6.47337 |
| GRIT-T2D+ | 0.93424 | 0.45201 | 0.04275 | 0.14444 | 0.24392 | 0.50134 | 0.17777 | 0.30528 | – | 0.37400 | 0.00701 | 0.15718 | -0.78135 | 6.47337 |

**Supplementary Table 16.** Baseline survival and mean component after recalibration in UK Biobank.

| | Women | | Men | |
|---|---|---|---|---|
| | **Baseline survival** | **Mean** | **Baseline survival** | **Mean** |
| **Models for CHD** | | | | |
| GRIT-CHD | 0.99406 | 6.89660 | 0.97526 | 5.62068 |
| GRIT-CHD+ | 0.99472 | 7.89214 | 0.97779 | 6.93821 |
| QRISK3 | 0.99479 | 1.54932 | 0.97678 | 1.57641 |
| PCE, untreated BP | 0.99536 | -29.36780 | 0.97894 | 61.13819 |
| PCE, treated BP | 0.98422 | -28.73394 | 0.95444 | 61.62163 |
| **Models for T2D** | | | | |
| GRIT-T2D | 0.99089 | 5.84887 | 0.98142 | 6.70147 |
| GRIT-T2D+ | 0.99262 | 5.79458 | 0.98329 | 7.00069 |
| QDiabetes | 0.99178 | 0.85458 | 0.98445 | 1.03729 |

**Supplementary Note 1.** Description of the CS-PRS-pipeline in FinnGen.

This CS-PRS pipeline represents work from the FinnGen analysis team, generated for the FinnGen data. The pipeline is also found at https://github.com/FINNGEN/CS-PRS-pipeline.

Pipeline to calculate PRS based on a list of sumstats.
Weights are calculated with PRScs: https://github.com/getian107/PRScs

## Rsid map
This step generates a mapping to/from rsid/chrompos based on data available at
ftp://ftp.ncbi.nih.gov/snp/organisms/human_9606_b151_GRCh38p7/VCF/00-All.vcf.gz.
`rsid_map.py` produces:
- finngen.rsid.map.tsv (rsid--> chrompos)

```
rs10            7_92754574
rs1000000       12_126406434
rs1000000219    13_95689463
```

- finngen.variants.tsv (chrompos --> ref/alt)

```
10_100000235    C    T
10_100000979    T    C
10_100001839    CAA  C
```

The first file is used throught the computation to go to/from rsid notation. The second is used to filter out variants that do not have the right alleles.

Also, if a rsid list is provided (e.g. hm3 rsids), it returns the subset of variants in the original bim file that match to those rsids:
- hm3.snplist

```
chr10_100000235_C_T
chr10_100002628_A_C
chr10_100004827_A_C
```

## Munging
PRScs automatically does some allele matching:
- the reference genome (1kg) only contains non ambigous variants :

```
cat snpinfo_1kg_hm3 | sed -E 1d |  cut -f 4,5 | awk '{print $1$2}' | sort | uniq
AC
AG
CA
CT
GA
GT
TC
TG
```

Also, in the parsing phase it checks for the ref/alt order and fixes the beta accordingly.

```
vld_snp = set(zip(vld_dict['SNP'], vld_dict['A1'], vld_dict['A2']))
```

ref_snp = set(zip(ref_dict['SNP'], ref_dict['A1'], ref_dict['A2'])) | set(zip(ref_dict['SNP'], ref_dict['A2'], ref_dict['A1'])) | \
set(zip(ref_dict['SNP'], [mapping[aa] for aa in ref_dict['A1']], [mapping[aa] for aa in ref_dict['A2']])) | \
set(zip(ref_dict['SNP'], [mapping[aa] for aa in ref_dict['A2']], [mapping[aa] for aa in ref_dict['A1']]))
sst_snp = set(zip(sst_dict['SNP'], sst_dict['A1'], sst_dict['A2'])) | set(zip(sst_dict['SNP'], sst_dict['A2'], sst_dict['A1'])) | \
set(zip(sst_dict['SNP'], [mapping[aa] for aa in sst_dict['A1'] if aa in ATGC], [mapping[aa] for aa in sst_dict['A2'] if aa in ATGC])) | \
set(zip(sst_dict['SNP'], [mapping[aa] for aa in sst_dict['A2'] if aa in ATGC], [mapping[aa] for aa in sst_dict['A1'] if aa in ATGC]))

comm_snp = ref_snp & vld_snp & sst_snp
with open(sst_file) as ff:
if (snp, a1, a2) in comm_snp:
...
beta_std = sp.sign(beta)*abs(norm.ppf(p/2.0))/n_sqrt
elif (snp, a2, a1) in comm_snp:
beta_std = -1*sp.sign(beta)*abs(norm.ppf(p/2.0))/n_sqrt
...

```
The final weights are printed based on the a1/a2 order of the reference panel (i.e. the EUR 1kg panel in this case).

PRScs does check for strand flip.

Our solution is therefore the following.

We build a rsid to chrom pos mapping from `ftp://ftp.ncbi.nih.gov/snp/organisms/human_9606_b151_GRCh38p7/VCF/00-All.vcf.gz` . This allows to move back and forth from/to rsid/chrompos notations and therefore to merge summary stats with different formats.

Then:
- in the munging phase we split the summary stats entries based on whether variants are identified by rsid or by some of chrom/pos notation
- the rsid file is filtered for rsids present in finngen. chrom and pos information are updated to finngen data
- the chrompos file is updated to have chrom and pos added (if provided, else extracted from variant id) and then lifted to build 38
- the two files are then merged to a FINNGEN chrom_pos_ref_alt notation, making sure that the variant exists in finngen data (checking for strand flip as well).

This produces a file with chrom and pos based on Finngen, but with A1/A2/OR/P based on the original data:

```
CHR SNP A1 A2 BP OR P
19 chr19_260912 G A 260912 0.9957872809136792 0.050031874722
19 chr19_261033 A G 261033 0.9957998422696626 0.0507241244322
19 chr19_266034 C T 266034 1.0053796666398893 0.150796052266
19 chr19_267039 C T 267039 0.9961140904000996 0.0691110781996
19 chr19_276245 T C 276245 0.995420944482037 0.0366293445837
19 chr19_277776 A G 277776 0.9964618752820534 0.119939685495
19 chr19_280299 C T 280299 0.9964396546231319 0.120269108388

```
19	chr19_281360	T	C	281360	0.9968755417956986	0.169347605744
19	chr19_288246	C	T	288246	0.9991125513478095	0.722369442562
19	chr19_288374	C	T	288374	1.0021901113140002	0.299346915861
```

## Weights

Weights are calculated using PRScs.
In order to run PRScs we then convert the file to rsids:
```
SNP	A1	A2	OR	P
rs8100066	G	A	0.9957872809136792	0.050031874722
rs8105536	A	G	0.9957998422696626	0.0507241244322
rs2312724	C	T	1.0053796666398893	0.150796052266
rs1020382	C	T	0.9961140904000996	0.0691110781996
rs12459906	T	C	0.995420944482037	0.0366293445837
rs11084928	A	G	0.9964618752820534	0.119939685495
rs11878315	C	T	0.9964396546231319	0.120269108388
rs7815	T	C	0.9968755417956986	0.169347605744
rs10409452	C	T	0.9991125513478095	0.722369442562
rs12981067	C	T	1.0021901113140002	0.299346915861
```

This guarantees that the beta is still correct,since it's based on the original summary stats. However, this way, we can "recycle" the munged data also for other reference panels, if needed in the future.

Then PRScs is run and weights are calculated only for the subset of variants shared across reference panel, summary stats and validation bim file (finngen).

```
19	rs8100066	260912	G	A	-2.438700e-05
19	rs8105536	261033	A	G	-1.608225e-05
19	rs2312724	266034	C	T	2.586432e-04
19	rs1020382	267039	C	T	8.532887e-06
19	rs12459906	276245	T	C	-3.629306e-05
19	rs11084928	277776	A	G	-3.484712e-05
19	rs11878315	280299	C	T	-6.442467e-06
19	rs7815	281360	T	C	-1.508200e-05
19	rs10409452	288246	C	T	2.060791e-05
19	rs12981067	288374	C	T	4.191780e-05
```

The weight file is converted to chrom_pos again through the finngen rsid/chrom_pos mapping,using the a1/a2 from the weights. However, now there is a double mismatch that needs to be fixed:
1) the weights were calculated based on the a1/a2 order of the reference data set
2) the output positions are based on the reference data set.

```
19	chr19_1208073_C_T	1208072 C	T	7.354914e-06
19	chr19_1218220_T_C	1218219 T	C	5.217805e-06
19	chr19_1220005_G_A	1220004 G	A	9.294323e-05
19	chr19_1221162_T_C	1221161 T	C	1.765576e-05
```

19 chr19_1226005_A_C 1226004 A C 8.979659e-05
19 chr19_1232559_C_T 1232558 C T 4.271571e-05
19 chr19_1238900_C_T 1238899 C T 2.828170e-05

```

In order to fix this, we replicate each entry, considering all possible permutations of the ref_alt in the variant id. This guarantees that at least one permutation is the matching Finngen variant. Also, the position is updated to the one in the id.

```
19 chr19_1208073_C_T 1208073C T 7.354914e-06
19 chr19_1208073_T_C 1208073C T 7.354914e-06
19 chr19_1208073_G_A 1208073C T 7.354914e-06
19 chr19_1208073_A_G 1208073C T 7.354914e-06
19 chr19_1218220_T_C 1218220T C 5.217805e-06
19 chr19_1218220_C_T 1218220T C 5.217805e-06
19 chr19_1218220_A_G 1218220T C 5.217805e-06
19 chr19_1218220_G_A 1218220T C 5.217805e-06
19 chr19_1220005_G_A 1220005G A 9.294323e-05
19 chr19_1220005_A_G 1220005G A 9.294323e-05
19 chr19_1220005_C_T 1220005G A 9.294323e-05
19 chr19_1220005_T_C 1220005G A 9.294323e-05
19 chr19_1221162_T_C 1221162T C 1.765576e-05
19 chr19_1221162_C_T 1221162T C 1.765576e-05
```

Now we have all elements in place:
- variants are identified with a Finngen ID
- the position is updated to finngen data
- the effect allele is still the original one
- weights are calcualted accordingly based on the effect allele

## Scores
Finally scores are calculate with `plink2 --sscore` which will only compute if the variant ids match, but still computing the score for the correct allele.
```
