# Supplementary material for: Integration of questionnaire-based risk factors improves polygenic risk scores for human coronary heart disease and type 2 diabetes
Source: Commun Biol. 2022 Feb 23;5:158. doi: 10.1038/s42003-021-02996-0 (PMC8866413; doi:10.1038/s42003-021-02996-0)
Supplement: Supplementary file 3 — Editorial Assessment Report [file 42003_2021_2996_MOESM3_ESM.pdf]

Contents of this report

- **Manuscript details**: overview of your manuscript and the editorial team.
- **Review synthesis**: summary of the reviewer reports provided by the editors.
- **Editorial recommendation**: personalized evaluation and recommendation from all 3 journals.
- **Annotated reviewer comments**: the referee reports with comments from the editors.
- **Open research evaluation**: advice for adhering to best reproducibility practices.

About the editorial process

Because you selected the **Nature Portfolio Guided Open Access option**, your manuscript was assessed for suitability in three of our titles publishing high-quality work across the spectrum of genetics research: *Nature Genetics, Nature Communications*, and *Communications Biology*. More information about Guided Open Access can be found here.

Collaborative editorial assessment

Your editorial team discussed the manuscript to determine its suitability for the Nature Portfolio Guided OA pilot. Our assessment of your manuscript takes into account several factors, including whether the work meets the **technical standard** of the Nature Portfolio and whether the findings are of **immediate significance** to the readership of at least one of the participating journals in the Nature Portfolio Guided Open Access genetics cluster.

Peer review

Experts were asked to evaluate the following aspects of your manuscript:

- **Novelty** in comparison to prior publications;
- **Likely audience** of researchers in terms of broad fields of study and size;
- **Potential impact** of the study on the immediate or wider research field;
- **Evidence** for the claims and whether additional experiments or analyses could feasibly strengthen the evidence;
- **Methodological detail** and whether the manuscript is reproducible as written;
- Appropriateness of the literature review.

Editorial evaluation of reviews

Your editorial team discussed the potential suitability of your manuscript for each of the participating journals. They then discussed the revisions necessary in order for the work to be published, keeping each journal's specific editorial criteria in mind.

Journals in the Nature portfolio will support authors wishing to transfer their reviews and (where reviewers agree) the reviewers' identities to journals outside of Springer Nature.

If you have any questions about review portability, please contact our editorial office at guidedoa@nature.com.

Manuscript details

Tracking number	Submission date	Decision date
GUIDEDOA-21-00157	21 June 2021	13 August 2021

**Title**	Improving polygenic risk assessment with simple questionnaire-based risk factors in coronary heart disease and type 2 diabetes	**Corresponding author**	Samuli Ripatti<br>**Affiliation:** University of Helsinki
**Preprint information**	There is no preprint available for this manuscript.	**Peer review type**	Single-blind

Editorial assessment team

**Primary editor**	**George Inglis**<br>**Home Journal:** *Communications Biology*, ORCID: 0000-0002-9069-5242<br>**Email:** **george.inglis@us.nature.com**
**Editorial team members**	**Ingrid Knarston**, *Nature Communications*, ORCID: 0000-0002-0932-8649<br>**Kyle Vogan**, *Nature Genetics*, ORCID: 0000-0001-9565-9665
**About your primary editor**	George received his PhD in Genetics and Molecular Biology from Emory University, where he studied mouse models of voltage-gated sodium channel dysfunction and epilepsy. He also has research experience in epigenomics and in vitro models of neuronal development. George joined the editorial team of *Communications Biology* in September 2020 and is based in the New York office.

Editorial assessment and review synthesis

**Editor's summary and assessment**	Here, the authors are interested in enhancing polygenic risk score (PRS) prediction for complex disorders like coronary heart disease (CHD) and type 2 diabetes (T2D). Using the FinnGen cohort for discovery and UK Biobank for validation, the authors develop a genomics-enhanced risk tool (GRIT) for both CHD and T2D, which incorporates risk metrics and lifestyle factors to improve predictive ability. The authors demonstrate that GRIT-CHD+ and GRIT-T2D+ each possess greater predictive ability compared to existing risk scores for each disorder (like QRISK3 or QDiabetes).<br><br>The editors jointly decided to send this manuscript out to review based on the improved utility of GRIT-CHD+ and GRIT-T2D+ over relevant risk scores. However, the fact that other studies have similarly demonstrated the benefits of integrating clinical data to improve risk prediction limited the novelty of this approach and prohibited further consideration by *Nature Genetics*. Similarly, the editors at *Nature Communications* had some concerns about the level advance over similar clinical risk models.
**Editorial synthesis of reviews**	The referees largely find the study to be well-designed, but there are some comments regarding the extent to which GRIT scores outperform existing alternatives, and the novelty of the study, which bolstered the initial concerns from the editors at *Nature Communications*. As best highlighted by Referee #3, there is a concern that GRIT may offer more limited accuracy when analyzing relevant clinical metrics, and Referee #2 also comments on the need to better demonstrate an additive effect of the PRS with clinical risk factors. Referees also commented on the generalizability of this dataset as a potential limitation.<br><br>While *Nature Genetics* and *Nature Communications* are unable to offer a revision, *Communications Biology* would be interested in considering a manuscript that (at a minimum) includes the following revisions:<br><br>**(1)** Please update analyses after accounting for ancestry (noted by Referee #3) and how differences in input weights or sources of validation data may impact the prediction model (Referee #2).<br><br>**(2)** As outlined by Referee #2, please demonstrate the predictive value of the PRS alone and simple risk factors alone, to highlight the utility of combining these components into a single score.<br><br>**(3)** Clearly state the limitations of GRIT, including the generalizability of this score (having utilized two European datasets) and potential tradeoffs between the OR vs. clinical metrics like the NRI (as best outlined by Referee #3).

Editorial recommendation

nature genetics	Revision not invited	As reflected in the feedback from Reviewers #1 and #3, we think the degree of conceptual advance and performance gain over previous studies in this area are not sufficient to meet our current editorial selection criteria.
nature communications	Revision not invited	Along with our editorial assessment, the feedback from Reviewer #1 and Reviewer #3 has bolstered our opinion that the advance and performance over existing approaches has not reached the level needed for publication at *Nature Communications*.
communications biology	Minor Revisions	As noted by the reviewers, improved analysis of potential statistical confounds, further demonstration of how GRIT scores compare to the PRS alone, and discussion of any limitations of this approach would be necessary for further consideration.

Next steps

Recommendation Summary: The manuscript and feedback from reviewers have been carefully considered by editors at all three journals. Based on this collective discussion, *Nature Genetics* and *Nature Communications* can no longer consider the manuscript due to concerns about the limited conceptual advance and improvement over existing approaches. However, the editors at *Communications Biology* would be especially interested in considering a manuscript that addresses reviewer concerns, as outlined in below.

- **Option 1:** Revise for consideration at *Communications Biology*
- **Option 2:** Revise for submission elsewhere

Revision

To follow our recommendation, please upload the revised manuscript, along with your point-by-point response to the reviewers' reports and editorial advice **using the link provided in the decision letter**.

Revision checklist

- Cover letter, stating to which journal you are submitting
- Revised manuscript
- Point-by-point response to reviews
- Updated **Reporting Summary** and **Editorial Policy Checklist**
- Supplementary materials (if applicable)

Submission elsewhere

Within the Nature Portfolio

Springer Nature provides authors with the ability to transfer a manuscript within the Nature Portfolio, without the author having to upload the manuscript data again. To use this service, please **follow the transfer link provided in the decision letter**. Note that any decision to opt in to *In Review* at the original journal is not sent to the receiving journal on transfer. You can opt in to *In Review* at receiving journals that support this service by choosing to modify your manuscript on transfer.

To a journal outside of Nature Portfolio

If you choose to submit your revised manuscript to a journal at another publisher, we can share the reviews with another journal outside of the Nature Portfolio if requested. You will need to request that the receiving journal office contacts us at guidedOA@nature.com. We have included editorial guidance below in the reviewer reports and open research evaluation to aid in revising the manuscript for publication elsewhere.

Annotated reviewer reports

The editors have included some additional comments on specific points raised by the reviewers below, to clarify requirements for publication in the recommended journal(s). However, please note that all points should be addressed in a revision, even if an editor has not specifically commented on them.

Reviewer #1	
**Reviewer #1**	This reviewer has not chosen to waive anonymity. The reviewer's identity can only be shared with representatives of an established journal editorial office.
**Reviewer #1 expertise**<br>Summarised by the editor	This reviewer has expertise in cardiovascular genomics.
**Editor's comments about this review**	While this reviewer provided an overall positive assessment of the manuscript, they do raise some concerns about novelty and generalizability that prohibited further consideration by *Nature Genetics* and *Nature Communications*.
**Reviewer #1 comments**	
**Overview**	The authors present straightforward and rigorously derived and presented polygenically-informed cardiometabolic disease prediction models. The models perform as-well to somewhat better than previously described scores with some interesting gains in net reclassification and detection of incident events. The authors state they are able to make these predictions via genetics + survey without the measure of clinical risk factors - but some of the survey questions are dependent upon the measurement of those risk factors (i.e. being on a statin or blood pressure medication). Regardless, as a research tool, which seems to be the intent of the authors, there are some advantages to the simplicity of the query. Overall, a well-presented derivation of a novel risk score with some benefits in research implementation.

Specific comments

#	Reviewer comment	Editorial comment

**1**	I would be interested to know if the GRIT-T2D score could contribute to CHD prediction.	Please elaborate on whether there might be cross-talk between the GRIT scores.
**2**	*Nature Communications* seems fine. There is nothing terribly novel here - the authors applied a PRS derivation tool to their cohort and validated in another (with inclusion of clinical risk). Nothing novel in terms of predictors and thus no great resultant gain in power. There are some nice details for model derivation - that could be a nice reference for the field.	While we appreciate the reviewer's input, the editors at *Nature Communications* feel that the limited conceptual advance prohibits further consideration of this manuscript.
**3**	The claims are fine but the authors are not claiming anything dramatic. The claim of the questions being survey based only are off-base as a clinical tool as you would need to have measured LDL or bp to have medication prescribed. But as a data gathering research tool the approach makes sense. I feel this is the likely intention, to help with data mining across cohorts.	
**4**	Independent replication is the gold standard. These are some very European cohorts - but they've made no claim to generalizability.	Please comment on any limitations regarding generalizability.
**5**	Pretty basic stuff but well presented with interesting utility. Certainly doesn't rise to the level of *Nature Genetics*. But it's a decent reference point for the field.	

Reviewer #2

**Reviewer #2**	This reviewer has not chosen to waive anonymity. The reviewer's identity can only be shared with representatives of an established journal editorial office.
**Reviewer #2 expertise**<br>Summarised by the editor	This reviewer has expertise in GWAS, PRS, and Mendelian randomization approaches.
**Editor's comments about this review**	This reviewer highlighted the need to better demonstrate an additive effect in combining clinical metrics and the PRS, and potential issues in the training and validation sets that should be addressed in a revision.

Reviewer #2 comments

**Overview**	Tamlander et al. have developed new genomics-enhanced risk tools (GRIT) for CHD and T2D by integrating genetic risk scores with simple risk factors from online questionnaires, without additional laboratory tests.<br>The authors have derived the prediction models in the FinnGen study, and have independently validated them in the UK Biobank. The models have shown comparable predictive performance to some well-known clinical risk scores (QRISK 3 and QDiabetes).

Specific comments

#	Reviewer comment	Editorial comment
1	**Lines 21-30:** The baseline models are PRS + age / PRS+age+BMI. Since the whole point of the paper is PRS + simple risk factors, I would also recommend showing the prediction of PRS alone and simple risk factors alone.	This point would be necessary for consideration in *Communications Biology*, and to best highlight the improvement of GRIT scores over baseline PRSs.
2	**Figure 2:** the authors have shown the performance of QRISK3 is worse than GRIT-CHD+ or even GRIT-CHD. From Supplementary Table 4, it seems all variables in QRISK3 except PRS were included in GRIT-CHD+ / GRIT-CHD. Is the higher prediction value of GRIT-CHD and GRIT-CHD+	*Communications Biology* would ask that you evaluate participants with these missing values, as suggested.

	mainly due to PRS? This should be discussed.	
**3**	**Supplementary Tables 2A-B & 3A-B:** The authors have shown the participant characteristics for excluded individuals, e.g. in Supp. Table 3B, a relatively large proportion of participants was excluded due to the missing HDL-C levels (43829/121113 = 36%). It might be worth exploring the characteristics of participants with missing values. Would imputation provide a more reasonable validation dataset compared with excluding missing values?	
**4**	The authors mentioned that the input weight samples partly overlap with FinnGen study and are independent of UKB. The effect on training the PRS model should be discussed.	
**5**	The validation UKB data for CHD and T2D are from different sources, eg. ICD10/9 hospital inpatient records, OPCS4, medication, general practice codes, and self-reported codes in 20002. They have different coverage of UKB participants, eg, general practice Read V2 and Read V3 clinical codes cover half UKB samples. Will this affect the validation step for GRIT - CHD and T2D?	For the sake of reproducibility, please clarify whether integration of these distinct data sources may impact the validation model.

Reviewer #3

**Reviewer #3**	This reviewer has not chosen to waive anonymity. The reviewer's identity can only be shared with representatives of an established journal editorial office.
**Reviewer #3 expertise** Summarised by the editor	Quantitative human genomics and polygenic risk scores
**Editor's comments about this review**	While this reviewer provided an overall positive assessment, they raised serious concerns about the novelty which, when considered with the feedback from Referee #1, prohibited further consideration by *Nature Genetics* and *Nature Communications*. This reviewer also raised several important points to improve the reproducibility of the study, and clarify the underlying Methods.

Reviewer #3 comments

**Overview**	This manuscript describes the combination of new PRS and clinical assessments for prediction of incident CAD and T2D. The paper is clearly written and comparatively exhaustive in explanation, though some holes remain in my view, as mentioned below. Positives include the use of PRS-CS to generate best-in-class PRS, out-of-sample validation in the complete British UKBB, and demonstration that blood pressure, cholesterol, and triglycerides as continuous traits improve joint modeling.

Specific comments

#	Reviewer comment	Editorial comment
**Major Concerns:**		
**1**	However, I have to say that I am struggling to see what is new here. Multiple studies cited in the manuscript (including one from the second author) and others have established both that PRS are strongly predictive of incident and prevalent CAD or T2D, and that at least for CAD the PRS add surprisingly little to the established clinical factors after middle age.	
**2**	The title of the manuscript implies that simple questionnaires are adequate to capture these clinical factors, which may be novel, but the strongest	

	incremental improvement is with BP, HDL and TG, which are not questionnaire-based measures.	
3	In my opinion the authors overstate the case for improvement due to PRS to a high degree. Although the GRIT scores are significantly higher than Baseline PRS+age+sex as well as PCE/QRISK3/QDiabetes in **Figure 2**, **Fig S3** places this in perspective: the increment is in no way clinically meaningful as the ROC almost overlay.	
4	The OR are also significantly higher, but the relevant clinical measure is the NRI, which is also improved by several percent BUT at the cost of a large number of reverses classifications. Improving the classification for 20 people while decrementing it for 15 results in a net improvement but at substantial cost.	
5	**Table 2** presents the numbers, but it is not transparent, and shows clearly that 10x to 20X non-cases are reclassified than cases.	
6	I think the message of the paper would be much clearer with a statement not just of NRI but of the number reclassified to achieve this net gain – something analogous to the number needed to treat.	Further discussion of the clinical utility or tradeoff of GRIT scores would be necessary for further consideration in *Communications Biology*. This point also ties in with the below comment regarding the applicability of GRIT scores.
7	A focus on the performance of PRS solely in the high risk (young, before onset of the clinical factors; female, normoweight) should provide a stronger statement of utility. The data presented seem to reaffirm that PRS have little added utility in moderate or high clinical risk individuals.	
**Minor Concerns:**		
8	**Table 1:** Numbers for Derivation CHD do not add up: 30,836+28,104 ≠ 61,878	
9	Does age refer to age at enrollment or incidence or endpoint?	

**10**	**Page 9, Line 12:** In the Statistical Analysis section, maximum follow-up time is stated as 10 years from baseline (enrollment?), but on the next page in the Results the median follow-up time was 15.3 years. Please explain.	
**11**	I understand the rationale for removing prevalent cases at Baseline, but can the authors estimate the impact on under-estimation of prevalence and PRS performance in a naïve (ideally, younger) population. The genetics should be stronger in the removed prevalent cases, but disease is also less prevalent.	
**12**	Please provide more details on the calibration: do not assume that readers understand the concepts of baseline hazard and mean component, or how they were used for recalibration. This is really important in my opinion since calibration is critical but poorly discussed or understood in the literature.	
**13**	Accordingly, I am concerned that the prevalence in FinnGen (Table 1) is ~3X greater for both traits in Finland than the UK. Quite apart from this being surprising to me, it must impact PRS assessment, but intuitively (correct me if I am wrong) in the opposite direction to that shown in **Fig 2**. Higher prevalence generally implies lower OR per Falconer, so applying the Finn OR to UKBB ought to underestimate prevalence, but apparently predicted risk us greatly elevated in the UKBB. Also, there does not appear to have been an adjustment for Finnish ancestry. These issues should be discussed for clarity.	
**14**	Reproducibility of methods is fine, but as I understand it few researchers have open access to Finnish databanks - though it can be arranged as a collaboration.	
**15**	I do not feel that all of the Supplementary Data is necessary, so in that sense they have gone overboard in promoting repeatability.	We would leave this point up to the authors' discretion.
**16**	Depositing the PRS-CS scores in the public database on publication is really good	Please note that *Communications Biology* does require the PRS to be deposited to the PGS Catalog prior to acceptance.

Open research evaluation

Data availability

Data availability statement

Nature Portfolio policies include a strong preference for research data to be archived in public repositories. For data types without specific repositories, we recommend that data are deposited in a generalist repository such as figshare or Dryad. More information about our data availability policy can be found here: https://www.nature.com/nature-portfolio/editorial-policies/reporting-standards#availability-of-data

See here for more information about formatting your Data Availability Statement: http://www.springernature.com/gp/authors/research-data-policy/data-availability-statements/12330880

More information about our data availability policy can be found here: https://www.nature.com/nature-portfolio/editorial-policies/reporting-standards#availability-of-data

Mandatory data deposition

Submission of DNA-sequencing data to a community-endorsed, public repository is mandatory for publication in a Nature Portfolio journal and is best practice for publication in any venue. Accession numbers must be provided in the paper. Examples of appropriate public repositories are listed below:

- GenBank
- Sequence Read Archive (WGS or WES data)
- The European Nucleotide Archive (ENA)

For genome-wide association studies, submission of the summary statistics to a community-endorsed, public repository is mandatory for publication in a Nature Portfolio journal and is best practice for publication in any venue. Accession numbers must be provided in the paper.

For this data type, we recommend submission to the NHGRI-EBI GWAS Catalog: https://www.ebi.ac.uk/gwas/

Submission of Polygenic Risk Score dataset to a community-endorsed, public repository is mandatory for publication in a Nature Portfolio journal and is best practice for publication in any venue. Accession numbers must be provided in the paper.

For this data type, we recommend submission to the PGS Catalog: https://www.pgscatalog.org/

For more information on mandatory data deposition policies at the Nature Portfolio, please visit: http://www.nature.com/authors/policies/availability.html#data

For a list of approved repositories for each mandatory data type, please visit: https://www.springernature.com/gp/authors/research-data-policy/repositories/12327124

Other data requests

All source data underlying the graphs and charts presented in the main figures must be made available as Supplementary Data (in Excel or text format) or via a generalist repository (eg, Figshare or Dryad). This is mandatory for publication in a Nature Portfolio journal, but is also best practice for publication in any venue.

Please note that **Fig 2** and **Supp Fig 2** require associated source data.

This journal strongly supports public availability of data and custom code associated with the paper in a persistent repository where they can be freely and enduringly accessed or as a supplementary data file when no appropriate repository is available. If data and code can only be shared on request, please explain why in your data Availability Statement, and also in the correspondence with your editor. For more information, please refer to https://www.nature.com/nature-research/editorial-policies/reporting-standards#availability-of-data

Please ensure that datasets deposited in public repositories are now publicly accessible, and that accession codes or DOI are provided in the "Data Availability" section. As long as these datasets are not public, we cannot proceed with the acceptance of your paper. For data that have been obtained from publicly available sources, please provide a URL and the specific data product name in the data availability statement. Data with a DOI should be further cited in the methods reference section.

Ethics

Because your study includes human participants, confirmation that all relevant ethical regulations were followed is needed, and that informed consent was obtained. This must be stated in the Methods section, including the name of the board and institution that approved the study protocol. Please also reiterate any relevant ethical statements for the UKBB.

Reporting and reproducibility

Please state in the legends how many times each experiment was repeated independently with similar results. This is needed for all experiments, but is particularly important wherever results from representative experiments (such as micrographs) are shown. If space in the legends is limiting, this information can be included in a section titled "Statistics and Reproducibility" in the methods section.

Nature Portfolio journals allow unlimited space for Methods. The Methods must contain sufficient detail such that the work could be repeated. It is preferable that all key methods be included in the main manuscript, rather than in the Supplementary Information. Please avoid use of "as described previously" or similar, and instead detail the specific methods used with appropriate attribution.

Statistics

Wherever statistics have been derived (e.g. error bars, box plots, statistical significance) the legend needs to provide and define the n number (i.e. the sample size used to derive statistics) as a precise value (not a range), using the wording "n=X biologically independent samples/animals/cells/independent experiments/n= X cells examined over Y independent experiments" etc. as applicable.

We strongly discourage deriving statistics from technical replicates, unless there is a clear scientific justification for why providing this information is important. Conflating technical and biological variability, e.g., by pooling technically replicates samples across independent experiments is strongly discouraged. (For examples of expected description of statistics in figure legends, please see the following https://www.nature.com/articles/s41467-019-11636-5 or https://www.nature.com/articles/s41467-019-11510-4).

Statistics such as error bars, significance and p values cannot be derived from n<3 and must be removed from all such cases.

All error bars need to be defined in the legends (e.g. SD, SEM) together with a measure of centre (e.g. mean, median). For example, the legends should state something along the lines of "Data are presented as mean values +/- SEM" as appropriate. All box plots need to be defined in the legends in terms of minima, maxima, centre, bounds of box and whiskers and percentile.

Please note that the measure of centre for the error bars needs to be defined in the legends of **Fig. 2a-b** and **Supplementary Fig. 4a-b.**

Data presentation

Please ensure that data presented in a plot, chart or other visual representation format shows data distribution clearly (e.g. dot plots, box-and-whisker plots). When using bar charts, please overlay the corresponding data points (as dot plots) whenever possible and always for n ≤ 10. (Please see the following editorial for the rationale behind this request and an example https://www.nature.com/articles/s41551-017-0079).

Please ensure that any figures in the Supplementary Information are limited to a single page. For instance, **Supplementary Figures 2a and 2b** should either be confined to a single page, or classified as distinct figures (e.g. **Supp. Fig 2-3**).

Our style does not allow for sub-tables (ex. **Supp. Table 2a, 2b**). Please characterize these tables as distinct items (ex. **Supp Table 2a-b** would become **Supp Tables 2-3**).

The quality of some of the figures (ex. **Fig. 1** and **Supp. Fig. 4**) appears to be quite low. If possible, we suggest replacing these with higher-resolution images.

Other notes

We have included as an attachment to the decision letter a version of your Reporting Summary with a few notes. This is mainly for your information, but we hope it is helpful when preparing your revised manuscript. If you decide to resubmit the manuscript for further consideration, please be sure to include an updated Reporting Summary.
