# Supplementary material for: Integration of questionnaire-based risk factors improves polygenic risk scores for human coronary heart disease and type 2 diabetes
Source: Commun Biol. 2022 Feb 23;5:158. doi: 10.1038/s42003-021-02996-0 (PMC8866413; doi:10.1038/s42003-021-02996-0)
Supplement: Supplementary file 4 — List of Additional Supplementary Information [file 42003_2021_2996_MOESM4_ESM.docx]

**Supplementary Data 1**Disease endpoint definitions in FinnGen and UK Biobank.

**Supplementary Data 2**Regularly taken blood-pressure-lowering, statin, second generation antipsychotic, erectile dysfunction, and oral or parenteral steroid prescription medications in UK Biobank datafield 20003.

**Supplementary Data 3**UK Biobank datafields used to calculate clinical risk scores.

**Supplementary Data 4**
Source data underlying the figures in the main text and Supplementary Information
