# Supplementary material for: Integration of questionnaire-based risk factors improves polygenic risk scores for human coronary heart disease and type 2 diabetes
Source: Commun Biol. 2022 Feb 23;5:158. doi: 10.1038/s42003-021-02996-0 (PMC8866413; doi:10.1038/s42003-021-02996-0)
Supplement: Supplementary file 9 — Reporting Summary [file 42003_2021_2996_MOESM9_ESM.pdf]

nature research

Corresponding author(s): Ripatti, S

Last updated by author(s): Dec 16, 2021

Reporting Summary

Nature Research wishes to improve the reproducibility of the work that we publish. This form provides structure for consistency and transparency in reporting. For further information on Nature Research policies, see our Editorial Policies and the Editorial Policy Checklist.

Statistics

For all statistical analyses, confirm that the following items are present in the figure legend, table legend, main text, or Methods section.

n/a	Confirmed	
☐	☒	The exact sample size (*n*) for each experimental group/condition, given as a discrete number and unit of measurement
☐	☒	A statement on whether measurements were taken from distinct samples or whether the same sample was measured repeatedly
☐	☒	The statistical test(s) used AND whether they are one- or two-sided<br>*Only common tests should be described solely by name; describe more complex techniques in the Methods section.*
☐	☒	A description of all covariates tested
☐	☒	A description of any assumptions or corrections, such as tests of normality and adjustment for multiple comparisons
☐	☒	A full description of the statistical parameters including central tendency (e.g. means) or other basic estimates (e.g. regression coefficient) AND variation (e.g. standard deviation) or associated estimates of uncertainty (e.g. confidence intervals)
☐	☒	For null hypothesis testing, the test statistic (e.g. *F*, *t*, *r*) with confidence intervals, effect sizes, degrees of freedom and *P* value noted<br>*Give P values as exact values whenever suitable.*
☒	☐	For Bayesian analysis, information on the choice of priors and Markov chain Monte Carlo settings
☒	☐	For hierarchical and complex designs, identification of the appropriate level for tests and full reporting of outcomes
☐	☒	Estimates of effect sizes (e.g. Cohen's *d*, Pearson's *r*), indicating how they were calculated

Our web collection on statistics for biologists contains articles on many of the points above.

Software and code

Policy information about availability of computer code

Data collection

No software was used for data collection.

Data analysis

We used following softwares for imputation, handling the genetic and phenotypic data:
Cromwell 61
Plink v2.00a2.3LM
BCFtools 1.7 and 1.9
Eagle 2.3.5
Beagle 4.1 (08Jun17.d8b)
R 3.6.0 and 4.1.1
R packages survminer_0.4.8, pROC_1.16.2
PRS-CS (https://github.com/getian107/PRScs)
The full genotyping and imputation protocol for FinnGen is described at dx.doi.org/10.17504/protocols.io.xbgfijw
The PRS-CS pipeline in FinnGen is described in Supplementary Note 1 and at https://github.com/FINNGEN/CS-PRS-pipeline.
The QRISK3 algorithm is described at https://qrisk.org/three/src.php
The QDiabetes-2018 algorithm is described at https://qdiabetes.org/src.php
The Pooled Cohort Equations algorithm is described at https://doi.org/10.1161/01.cir.0000437741.48606.98
The FINDRISC algorithm is described at https://doi.org/10.2337/diacare.26.3.725
For data transformations, visualization, and plotting of the results, we used R 3.6.0 (in UK Biobank) and R 4.1.1 (FinnGen).

For manuscripts utilizing custom algorithms or software that are central to the research but not yet described in published literature, software must be made available to editors and reviewers. We strongly encourage code deposition in a community repository (e.g. GitHub). See the Nature Research guidelines for submitting code & software for further information.

Data

Policy information about availability of data

All manuscripts must include a data availability statement. This statement should provide the following information, where applicable:

- Accession codes, unique identifiers, or web links for publicly available datasets
- A list of figures that have associated raw data
- A description of any restrictions on data availability

The Finnish biobank data can be accessed through the Fingenious® services (web link: https://site.fingenious.fi/en/, email: contact@finbb.fi) managed by FINBB. The UK Biobank resource is available to bona fide researchers for health-related research in the public interest at https://www.ukbiobank.ac.uk/researchers/. The GWAS summary statistics used for constructing our PRSs are available at http://www.cardiogramplusc4d.org/data-downloads/ and https://diagram-consortium.org/downloads.html. The weights for our PRSs are available at PGS Catalog (pgs-info@ebi.ac.uk) at https://www.pgscatalog.org/score/PGS001780/ and https://www.pgscatalog.org/score/PGS001781/. LD reference panels constructed using the 1000 Genomes Project (https://doi.org/10.1038/nature15393) phase 3 samples can be downloaded at https://github.com/getian107/PRScs. Supplementary Data 4 contains the raw data underlying the figures in the main text and Supplementary Information.

Field-specific reporting

Please select the one below that is the best fit for your research. If you are not sure, read the appropriate sections before making your selection.

☒ Life sciences ☐ Behavioural & social sciences ☐ Ecological, evolutionary & environmental sciences

For a reference copy of the document with all sections, see nature.com/documents/nr-reporting-summary-flat.pdf

Life sciences study design

All studies must disclose on these points even when the disclosure is negative.

Sample size

The study is based on FinnGen Data Freeze 7 (total n = 309,154 individuals, final sample size n = 61,878 individuals with 3,536 incident cases in CHD derivation dataset and n = 69,159 individuals and 4,892 incident cases in T2D derivation dataset) and UK Biobank British ancestry subset (total n = 343,672 individuals, final sample size n = 242,687 individuals with 4,469 incident cases in CHD validation dataset and n = 121,113 individuals with 2,544 incident cases in T2D validation dataset). The pre-established exclusion criteria are described in detail in the Methods and below. We have shown that earlier data freezes (https://doi.org/10.1038/s41591-020-0800-0; https://doi.org/10.1038/s41467-020-19966-5) with smaller sample size have been sufficient for polygenic risk score analyses of common diseases.

Data exclusions

Exclusion of samples and variants was based on standard guidelines and quality control procedures. The pre-established exclusion criteria are described in detail in the Methods. Study participants who withdrew consent have been excluded.

FinnGen participants recruited after 2016 and/or aged <30 or >75 (n = 213,972) were excluded to have representative estimates in the derivation analyses.

UK Biobank participants with non-British ancestry were excluded based on genetically inferred ancestry. The British ancestry subset included 343,672 unrelated individuals.

Participants were excluded if they had prevalent cardiovascular disease (n = 10,471 individuals in FinnGen; n = 24,133 individuals in UK Biobank) or used statin medication (n = 8,839; n = 38,632) at baseline in CHD analyses or had prevalent diabetes (n = 7,877; n = 7,722) in T2D analyses. Participants with missing data on predictors needed to derive and calculate our new risk tools or calculate the established clinical risk scores (with the exception for smoking frequency in UK Biobank) were also excluded in CHD analyses (n = 20,413 individuals in FinnGen; n = 38,220 individuals in UK Biobank) and T2D analyses (n = 20,413; n = 31,503).

Replication

To benchmark our risk models, we externally validated them in the UK Biobank in 242,687 participants (CHD analyses) and 121,113 participants (T2D analyses) and compared them to established clinical risk scores (QRISK3 and Pooled Cohort Equations for CHD, FINDRISC and QDiabetes-2018 for T2D) by using their previously published weights. For reclassification analyses, we recalibrated the risk scores (excluding FINDRISC) by estimating and integrating the baseline hazard and mean component from the UK Biobank validation datasets to the linear predictor alongside the original beta coefficients, as detailed in the manuscript and Supplementary Information.

Randomization

The study does not include allocation of participants to different experimental groups. Due to the observational nature of the study and the FinnGen dataset, randomization was not applicable

Blinding

No blinding was relevant for this observational study.

Reporting for specific materials, systems and methods

We require information from authors about some types of materials, experimental systems and methods used in many studies. Here, indicate whether each material, system or method listed is relevant to your study. If you are not sure if a list item applies to your research, read the appropriate section before selecting a response.

Materials & experimental systems

n/a	Involved in the study
☒	☐ Antibodies
☒	☐ Eukaryotic cell lines
☒	☐ Palaeontology and archaeology
☒	☐ Animals and other organisms
☐	☒ Human research participants
☒	☐ Clinical data
☒	☐ Dual use research of concern

Methods

n/a	Involved in the study
☒	☐ ChIP-seq
☒	☐ Flow cytometry
☒	☐ MRI-based neuroimaging

Human research participants

Policy information about studies involving human research participants

Population characteristics

This study included participants from FinnGen Data Freeze 7 and UK Biobank. All individuals used in this study were of European ancestry.

The FinnGen CHD derivation dataset included 61,878 individuals, of which 45.4% were women; mean (SD) age for women was 51.3 (10.8) years and for men, 53.4 (10.7) years. The FinnGen T2D derivation dataset included 69,159 individuals, of which 43.8% were women; mean (SD) age for women was 52.4 (11.1) years and for men, 54.6 (10.8) years.

The UK Biobank CHD validation dataset included 242,687 individuals, of which 56.6% were women; mean (SD) age for women was 56.4 (7.9) years and for men, 56.3 (8.1) years. The UK Biobank T2D validation dataset included 121,113 individuals, of which 53.8% were women; mean (SD) age for women was 56.9 (7.9) years and for men, 57.3 (8.1) years.

The key characteristics of the study participants are described in the main text, Table 1, and Supplementary Tables 7–11.

Recruitment

This study included participants from FinnGen and UK Biobank.

FinnGen: Random sample of subjects from Finnish population-based and clinical biobanks. A proportion of FinnGen was ascertained through hospital biobanks and disease-based collections, but our decision to exclude participants recruited before 2017 (to ensure adequate follow-up) resulted in that the majority of included FinnGen participants were from population-based cohorts (Supplementary Table 1), which have also previously been widely used for building and testing risk assessment tools for CHD and T2D (https://doi.org/10.1038/s41591-020-0800-0; https://doi.org/10.1016/S0140-6736(10)61267-6; https://doi.org/10.1093/ije/dyx239; https://doi.org/10.2337/diacare.26.3.725; https://doi.org/10.1097/01.hjr.0000174793.31812.21). A small number of overlapping input weight samples for the PRS-CHD (a maximum of 1,208 controls free of CHD from the FINRISK 1997-2007 cohorts within the FinnGen study; https://doi.org/10.1038/ng.3396) might have slightly inflated the effect size of polygenic risk in the joint modeling in the derivation analyses. The potential downstream effect of this would, however, be decreased performance in the validation data, instead of inflated effects.

UK Biobank: The UK Biobank is a prospective cohort study that recruited over 500,000 participants from the United Kingdom between 2006 and 2010. Approximately 9.2 million individuals aged 40-69 years who lived within 25 miles of one of the 22 UK Biobank assessment centers in England, Wales, and Scotland were invited to enter the cohort, and 5.5% of the invited individuals completed the UK Biobank baseline assessment. Age at baseline ranged from 40 to 69. At baseline, individuals completed extensive questionnaires and an interview by a trained nurse about sociodemographic, lifestyle, and health-related factors, along with a range of physical and biomarker measurements. Despite a very low response rate resulting in oversampling of healthy individuals in UK Biobank, the risk factor associations (effect sizes) have been considered to be generalizable (https://doi.org/10.1093/aje/kwx246; https://doi.org/10.1136/bmj.m131).

Ethics oversight

Patients and control subjects in FinnGen provided informed consent for biobank research, based on the Finnish Biobank Act. Alternatively, separate research cohorts, collected prior the Finnish Biobank Act came into effect (in September 2013) and start of FinnGen (August 2017), were collected based on study-specific consents and later transferred to the Finnish biobanks after approval by Fimea (Finnish Medicines Agency), the National Supervisory Authority for Welfare and Health. Recruitment protocols followed the biobank protocols approved by Fimea. The Coordinating Ethics Committee of the Hospital District of Helsinki and Uusimaa (HUS) statement number for the FinnGen study is Nr HUS/990/2017.

The FinnGen study is approved by Finnish Institute for Health and Welfare (permit numbers: THL/2031/6.02.00/2017, THL/1101/5.05.00/2017, THL/341/6.02.00/2018, THL/2222/6.02.00/2018, THL/283/6.02.00/2019, THL/1721/5.05.00/2019, THL/1524/5.05.00/2020, and THL/2364/14.02/2020), Digital and population data service agency (permit numbers: VRK43431/2017-3, VRK/6909/2018-3, VRK/4415/2019-3), the Social Insurance Institution (permit numbers: KELA 58/522/2017, KELA 131/522/2018, KELA 70/522/2019, KELA 98/522/2019, KELA 138/522/2019, KELA 2/522/2020, KELA 16/522/2020, Findata THL/2364/14.02/2020 and Statistics Finland (permit numbers: TK-53-1041-17 and TK/143/07.03.00/2020 (earlier TK-53-90-20).

The Biobank Access Decisions for FinnGen samples and data utilized in FinnGen Data Freeze 7 include: THL Biobank BB2017_55, BB2017_111, BB2018_19, BB_2018_34, BB_2018_67, BB2018_71, BB2019_7, BB2019_8, BB2019_26, BB2020_1, Finnish Red Cross Blood Service Biobank 7.12.2017, Helsinki Biobank HUS/359/2017, Auria Biobank AB17-5154 and amendment #1 (August 17 2020), Biobank Borealis of Northern Finland_2017_1013, Biobank of Eastern Finland 1186/2018 and amendment 22 § /2020, Finnish Clinical Biobank Tampere MH0004 and amendments (21.02.2020 &

06.10.2020), Central Finland Biobank 1-2017, and Terveystalo Biobank STB 2018001.

The use of UK Biobank data does not require an ethics approval statement. UK Biobank data are available through a procedure described at http://www.ukbiobank.ac.uk/using-the-resource/. This project was conducted with permission of the UK Biobank Resource under application no. 22627. All participants gave electronic signed consent.

Note that full information on the approval of the study protocol must also be provided in the manuscript.
